# Molecular Design Considerations for Azobenzene Anolytes

**DOI:** 10.1021/acsomega.5c05073

**Published:** 2025-06-12

**Authors:** Ananya Banik, Uddalak Sengupta, Haley Hughes, Palani Sabhapathy, Burcu Gurkan, Emily B. Pentzer, David C. Powers

**Affiliations:** † 14736 Texas A&M University, Department of Chemistry, College Station, Texas 77843, United States; ‡ 2546 Case Western Reserve University, Department of Chemical and Biomolecular Engineering, Cleveland, Ohio 44106, United States

## Abstract

Realization of high-density batteries requires the development of anolytes that display highly negative reduction potentials, solubility, and persistence in the charged state. Azobenzenes have garnered interest as potential anolytes for redox flow batteries. Here, we report the synthesis of a family of substituted azobenzene derivatives and evaluation of their solution-phase electrochemical properties. Systematic synthetic derivatization of this scaffold allows (1) access to anolytes of varying solubility, including intrinsically liquid derivatives that represent potential high-density charge carriers; (2) systematic variation of the reduction potential, and in some cases redox inventory, that provides azobenzenes with highly negative reduction potentials; and (3) control of the lifetime of the azobenzene radical anions that result from one-electron reduction. Electrokinetic experiments demonstrated that fast electron transfer occurs for all derivatives examined. Spectroscopic characterization of monoreduced azobenzene derivatives establishes that decomposition of the azobenzene radical anion proceeds via bimolecular disproportionation. Together, these results provide an experimental basis for the optimization of azobenzene anolytes for electrochemical storage applications, including redox flow batteries.

## Graphical abstract

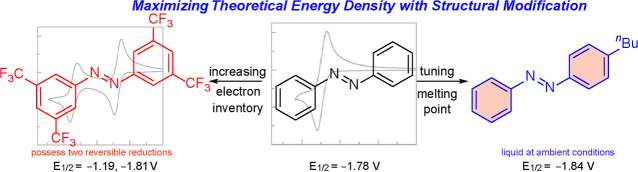

## Introduction

The intermittency of many sustainable energy sources requires advances in storage technology to manage the energy demands of modern society.^1–3^ Enormous progress in Li-ion technology and emerging platforms based on other electropositive metals, e.g., Na and Mg, provide high-power-density storage for distributed applications.^4,5^ Challenges in grid-scale storage have motivated extensive research for the development of redox flow batteries (RFBs), which store electrical potential in two liquid-phase electrolytes (i.e., anolyte^6^ and catholyte^7–9^ ).^10^ The storage capacity of RFBs depends on the concentration and volume of the charge-carrying solutions, while the power density depends on how rapidly the redox couple is discharged and the voltage difference between the anolyte and catholyte.^11^ Aqueous flow batteries^12–19^ have been extensively investigated but have inherent limitations to energy density due to the narrow electrochemical window of water.^20–22^ Nonaqueous organic redox flow batteries (NAORFBs) offer a potential solution to these challenges by expanding the potential window that can be accessed.^23^ Further, the inherent tunability of charge carriers based on organic small molecules provides opportunities to optimize the chemical and physical properties of the anolyte and catholyte to access high-density energy storage systems.^23^

NAORFBs encounter their own set of challenges, including (1) decomposition of electrolytes in the charged state (i.e., capacity fade),^24–26^ (2) potential chemical reactions between the anolyte and catholyte,^27,28^ (3) limited solubility of the molecular electrolytes, which limits energy density,^29^ and (4) loss of power density due to Ohmic drop.^28,29^ Among anolytes that have been studied for NAORFBs (Figure 1a),^29–32^ azobenzene derivatives show promise due to (1) ease and modularity of available synthetic methods, (2) stability of the azobenzene radical anions, (3) high charge/molecular weight ratio, and (4) opportunities for derivatization of azobenzene to impact solubility and redox potential (Figure 1b).^33–39^ Yu and co-workers demonstrated a NAORFB based on azobenzene (Ph-N=N-Ph).^33^ While the original system displayed good performance using *N*,*N*-dimethylformamide (DMF) as a solvent and lithium bis­(trifluoromethanesulfonyl)­imide (LiTFSI) as a supporting electrolyte, solubility limitations of azobenzene in the electrolyte limit the energy density of the resulting NAORFB, and poor solubility in other common organic solvents prevents systematically optimizing performance. Moreover, available NAORFB systems rely on one-electron reduction of azobenzenes, whereas two-electron reduction is also possible and doubles the gravimetric density of storage; instability of the doubly reduced species has thus far prevented utilization of both potential electron equivalents. Detailed understanding of the structure–property relationships that underpin azobenzene solubility, redox inventory, and reduction potential is critical to rational design of new, more highly performing NAORFBs (Figure 1c).

**Figure 1:**
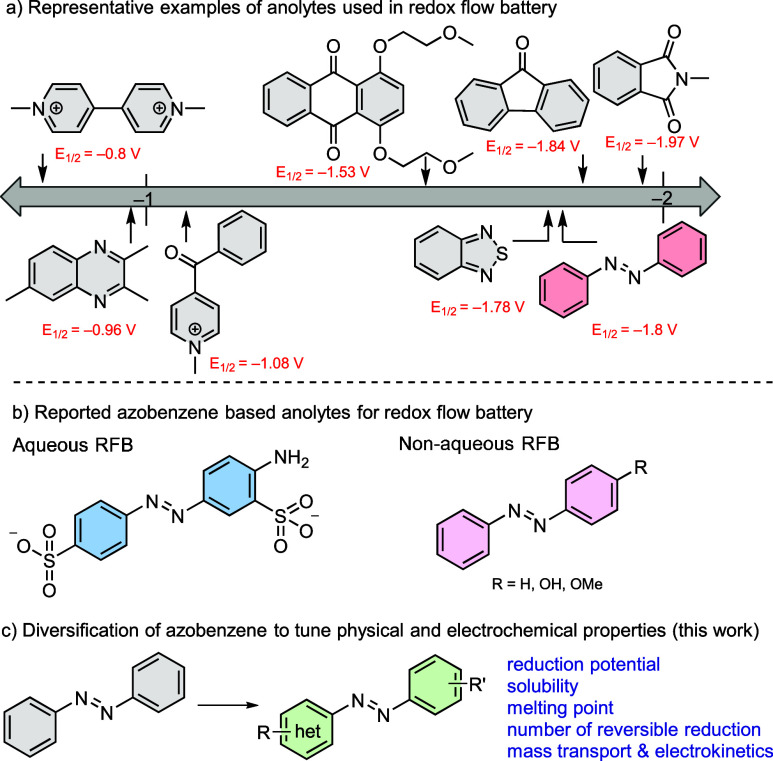
(a) Collection of molecular architectures that have been investigated as anolytes for nonaqueous redox flow batteries (NAORFBs) organized by one-electron reduction potential. (b) Peripheral substitution of azobenzenes has enabled application of these molecules as anolytes in aqueous and nonaqueous RFBs. (c) Here, we describe the impact of molecular structure on the electrochemistry, electrokinetics, solubility, and physical properties of functionalized azobenzenes.

Here, we report the synthesis and characterization of a library of azobenzene derivatives that allow the electrochemical properties, solubility, lifetimes of charged states, and redox inventory to be evaluated. The family of azobenzenes prepared displays a linear dependence of the reduction potential on the electronic demand of peripheral substituents. Regardless of the substitution pattern, the obtained azobenzenes display fast electron transfer kinetics and rapid diffusion under electrochemical conditions. The solubility and melting points of the azobenzenes vary significantly as a function of structure, with some derivatives being liquid under ambient conditions and having miscibility in common organic solvents. Spectroscopic characterization of the azobenzene radical anion provided the opportunity to evaluate decomposition mechanisms related to potential fade with these anolytes, and in some cases, highly reversible two-electron reduction processes could be validated. This study delineates the impact of the molecular structure of the electrochemical and physical properties that are critical to NAORFB performance.

## Results and Discussion

The energy density of an RFB is defined by eq 1:

1 $$E=\frac{NC_{a}FV}{\mu_{V}}$$

where *E* = system energy density, *N* = the number of electrons involved in the redox reaction, *F* = Faraday constant (26.8 A h mol^–1^), *C*_a_ = lower concentration of two active redox species, *V* = voltage of the cell, and μ_v_ = volume factor.^23^ Thus, in concept, high-energy RFBs can be realized by (1) increasing the solubility and thus the maximum concentration (i.e., *C*_a_) of the anolyte and/or catholyte, (2) increasing the potential difference between catholyte and anolyte (V), and (3) maximizing the electron inventory of the redox active species (i.e., multielectron electrolytes to increase N). Furthermore, chemical stability (i.e., long lifetimes) of the charged state of the electrolytes is critical to minimize capacity fade during battery cycling, and rapid mass transport and electron transfer kinetics during charging and discharging allow full use of the redox active species in solution. We reasoned the azobenzene derivatives represent an attractive platform to simultaneously optimize these features given the (1) highly negative intrinsic potential for electron injection (e.g., −1.78 V vs. Fc^+^/Fc for unsubstituted azobenzene), (2) the extremely modular synthesis of these compounds that can accommodate significant peripheral functionalization, and (3) the potential for multielectron chemistry within appropriately substituted azobenzenes.

### Synthesis and Electrochemical Characterization

A family of azobenzenes (**1a**–**1s**) was prepared by reaction of nitrosobenzene and appropriately substituted aniline derivatives;^40–44^ see Supporting Information for synthetic details. This scalable synthetic route commences with easily available starting materials and provides access to azobenzene derivatives bearing both electron-donating and electron-withdrawing substituents in analytical purity following a single-step chromatographic purification. Notably, many of the azobenzene derivatives described here represent new molecular targets, and even for those derivatives known, detailed investigations of the structure–property relationships that underpin electrochemistry, electrokinetics, and physical properties have not been described.

We evaluated the reduction chemistry of compound **1a** using a 5 mM acetonitrile (MeCN) solution with 0.1 M TBAPF_6_ as the supporting electrolyte using glassy carbon as the working electrode and Pt as the counter electrode. Under these conditions, **1a** shows reversible one-electron reduction with half-wave potential, *E*_1/2_ = –1.46 V vs. Fc^+^/Fc (Figure S1) with a peak current ratio (*i*_pa_/*i*_pc_) = 0.99. In addition, an irreversible reduction wave was observed at *E*_p_ = –2.02 V, which we attribute to a second reduction of the azobenzene derivative.^33^ Compound **1a** was chosen for optimization of the electrochemical parameters due to its ease of preparation (82% yield) and solubility in most organic solvents. The reversibility of the first reduction event displayed significant solvent dependence: While reversibility (*i*_pa_/*i*_pc_ = 0.99) was also observed in *N*,*N*-dimethylformamide (DMF), quasi-reversible reduction waves were measured in dichloromethane (CH_2_Cl_2_; *i*_pa_/*i*_pc_ = 0.94), 1,2-dichloroethane (DCE; *i*_pa_/*i*_pc_ = 0.72), and tetrahydrofuran (THF; (*i*_pa_/*i*_pc_ = 0.86). We also examined the impact of the supporting electrolyte on the electrochemistry of **1a**: Varying the electrolyte from TBAPF_6_, TBABF_4_, TBABr, and TBAClO_4_ had a negligible impact on the observed electrochemical reversibility (Figures S2 and S3). The electrochemical stability of **1a** under the optimized conditions (MeCN with 0.1 M TBAPF_6_) was tested by cycling 250 times between −2.00 and 0.00 V vs. Fc^+^/Fc. No impact on the shape or reversibility was observed in the cyclic voltammogram; further, cycling in the presence of ferrocene had no impact on the electrochemistry of **1a** (Figures S4 and S5).

With the optimized electrochemical conditions for **1a** in hand, we examined the impact of the azobenzene structure on the reductive electrochemistry by examining cyclic voltammograms of compounds **1b**–**1s** (Figure 2a); Table 1 summarizes the electrochemical properties of **1**. We observed that the introduction of electron-withdrawing substituents, such as –CN (**1d**), –COMe (**1b**), –COPh (**1c**), –CF_3_ (**1e**), and –Ph (**1f**), at the para position resulted in a less negative reduction potential by 310 mV, 250 mV, 230 mV, 210 mV, and 60 mV, respectively. Additionally, 4-nitroazobenzene (**1g**) (*E* ^1^_1/2_ = –1.24 V and *E* ^2^_1/2_ = –1.59 V vs. Fc^+^/Fc) and 3,3′,5,5′-tetra­(trifluoromethyl)­azobenzene (**1h**) (*E* ^1^_1/2_ = –1.19 V and *E* ^2^_1/2_ = –1.81 V vs. Fc^+^/Fc) each display two reversible reduction events, suggesting that highly electronegative substituents (–NO_2_ and –CF_3_) can stabilize the doubly reduced form. In contrast, azobenzenes bearing electron-donating substitution of 4-*n*-butyl (**1i**), *t*-butyl (**1j**), –OMe (**1k**), and–NMe_2_ (**1L**), as well as 4,4′-dimethoxy (**1m**) and 2,6-diisopropyl (**1n**), possess a more negative reduction potential of −1.84 V, −1.87 V, −1.87 V, −1.98 V, −1.98 V, and −2.00 V, respectively. Thus, the addition of electron-donating substituents increases the working potential (i.e., the potential difference between the catholyte and anolyte) between 60 and 220 mV compared to unsubstituted azobenzene.

**Figure 2:**
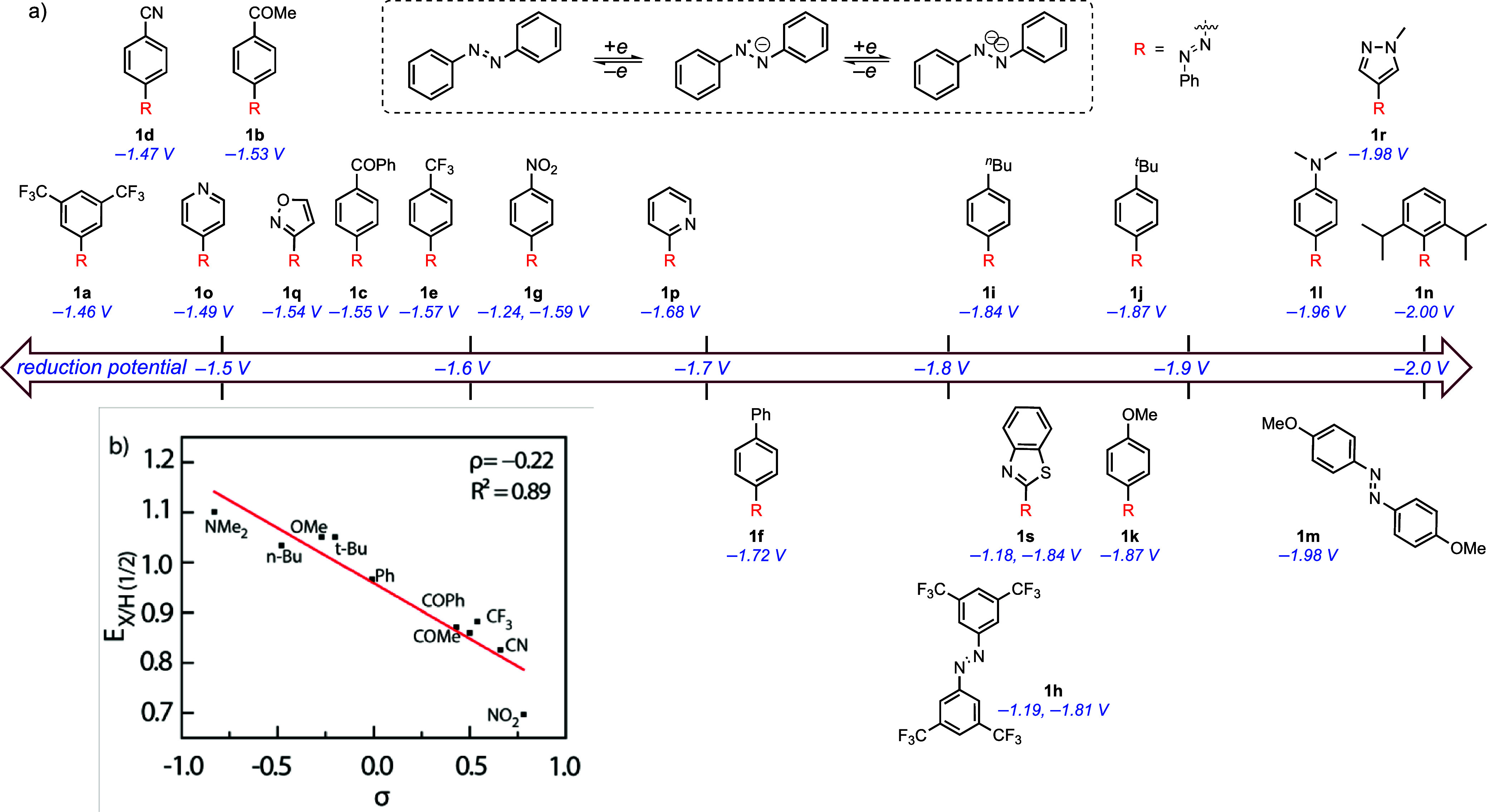
(a) Summary of electrochemical data of azobenzenes **1a**–**1s**. Cyclic voltammograms were measured using 0.005 M MeCN solutions (Fc^+^/Fc was used as a reference and 0.1 M TBAPF_6_ was used as a supporting electrolyte). (b) Hammett plot generated from between *E*_*X*/H(1/2)_ vs. Hammett parameter (σ_p_) for PhN = N–C_6_H_4_-4-X.

**Table 1:** Summary of the Electrochemical, Physical, and Spectroscopic Properties of **1**

compound	*E*^1^_1/2_ (V)	*i*_pa1_/*i*_pc1_	*E*^2^_1/2_ (V)	*i*_pa2_/*i*_pc2_	λ_max_ (nm)	melting range (°C)	MeCN solubility (M) (density (g/mL))
**1a**	–1.46	0.99			451	128–129	0.06
**1b**	–1.53	0.78			452	115–116	0.20
**1c**	–1.55	0.80			452	110–111	0.16
**1d**	–1.47	0.94			455	122–124	0.31
**1e**	–1.57	0.92			448	98–99	0.34
**1f**	–1.72	0.97			450	155–156	0.06
**1g**	–1.24	0.86	–1.59	0.82	458	133–134	0.41
**1h**	–1.19	0.96	–1.81	0.95	454	128–129	0.06
**1i**	–1.84	0.94			448	<23	miscible (ρ = 1.01)
**1j**	–1.87	0.90			449	54–55	2.46
**1k**	–1.87	0.81			436	58–59	4.10
**1l**	–1.96	0.50			412	119–120	0.25
**1m**	–1.98	0.73			357	165–167	0.08
**1n**	–2.00	0.60			457	<23	miscible (ρ = 0.99)
**1o**	–1.49	0.99			453	101–102	0.75
**1p**	–1.68	0.98			450	36–38	1.90
**1q**	–1.54	0.86			438	79–80	3.77
**1r**	–1.98	0.62			420	58–60	3.30
**1s**	–1.18	0.84	–1.84	0.69	367	149–150	0.16

The obtained electrochemical data for substituted asymmetric azobenzene derivatives are well correlated with the Hammett σ parameters: A plot of the half-wave potential for the first reduction of various 4-substituted azobenzene (*E*_*x*(1/2)_) normalized by the half-wave potential of unsubstituted azobenzene (*E*_H(1/2)_) versus the Hammett substituent constant (σ) is linear with ρ = – 0.22 (Figure 2b). The electrochemical reversibility of the azobenzene derivatives included in this plot, evaluated by the ratio of *i*_pa_/*i*_pc_, varies significantly across the trendline, with electron-rich azobenzenes displaying relatively poor reversibility (*i*_pa_/*i*_pc_ = 0.50 for **1l** and 0.81 for **1k**) and electron-deficient derivatives displaying highly reversible reduction chemistry (*i*_pa_/*i*_pc_ = 0.94 for **1d** and 0.92 for **1e**). Despite the differences in reversibility for different substituents, the linearity in the plot suggests that the reduction potentials can be evaluated accurately.^45,46^

The electrochemistry of heteroaryl-substituted azobenzenes **1o**–**1s** was also evaluated. Incorporation of heteroaryl groups, including pyridyl, isoxazole, *N*-methyl pyrrole, *N*-methyl pyrazole, and benzothiazole, yielded azobenzenes with reduction potentials between −1.49 V and −1.98 V (Figure 2a) and moderate to excellent redox reversibility (*i*_pa_/*i*_pc_ from 0.62 (**1r**) to 0.99 (**1o**)). Notably, 2-(phenyldiazenyl)­benzothiazole shows two reversible electrochemical reduction events at −1.18 and −1.84 V.

### Physical Properties of the Azobenzene Derivatives

Increasing the solubility of the redox-active materials can increase the energy density of the flow battery. Unsubstituted azobenzene shows a solubility of <1 M in MeCN,^33^ which is not practical for high-energy-density flow batteries. Systematic improvement in the solubility of charge carriers would translate to an increased energy density in the resulting battery; indeed, in the limit of solubility, one might envision a solvent-free liquid electrolyte. With access to a large family of azobenzenes with well-defined electrochemistry, we sought to establish the solubility of these molecules in organic solvents. In particular, given the low molar mass of azobenzene, we reasoned this to be an attractive scaffold to examine structure/solubility relationships.

The melting points of the azobenzenes and their solubilities in MeCN are listed in Table 1. As described above, unsubstituted azobenzene has a solubility of <1 M in MeCN and a melting point of ∼67 °C. Introduction of various functional groups markedly increased the melting point of the azobenzene derivatives, as seen for trifluoromethyl (**1a**, **1e**, and **1h**; 128, 122, and 133 °C, respectively), keto (**1b** and **1c**; 115 and 110 °C, respectively), cyano (**1d**, 122 °C), phenyl (**1f**, 155 °C), nitro (**1g**, 128 °C), and *N*,*N*-dimethyl (**1l**, 119 °C) groups. In contrast, alkyl (**1i**, **1j**, and **1n**; <23, 54, and <23 °C, respectively) or alkoxy (**1k**, 58 °C) substituents lower the melting point compared to azobenzene itself. The lowered melting points of **1i**, **1j**, and **1n** may result from the flexibility and rotational freedom of the alkyl substituents and thus less efficient packing of the azobenzene core.^47,48^ Enhanced solubility of the azobenzene derivatives in MeCN was observed in the case of alkyl and alkoxy substitution, with a maximum solubility of 4.1 M in the case of **1k**. A steady decrease in the solubility was noted for trifluoromethyl, ketone, cyano, phenyl, nitro, and *N*,*N*-dimethyl substituents. Anecdotally, azobenzene solubility is negatively correlated with the melting point (Figure S6): the azobenzene derivatives with high melting points display a low solubility, and the azobenzene derivatives with lower melting points have high solubility in MeCN.^49,50^

Remarkably, 4-*n*-butyl-substituted azobenzene (**1i**) is liquid at ambient conditions and completely miscible in organic solvents including hexanes, toluene, MeCN, CH_2_Cl_2_, and DMF (Figure 3). The half-wave potential of **1i** is −1.84 V, which is 60 mV more negative than that of unsubstituted azobenzene, and the ratio of anodic to cathodic peak current is 0.94. Further, the electrochemical cyclability of **1i** indicates that the molecule undergoes reversible electrochemical reduction even after 250 cycles (Figures S7 and S8), without any loss in reversibility. Moreover, the high-concentration electrochemical performance test shows that the cyclic voltammogram of **1i** is reversible upon increasing the concentration up to at least 2 M in acetonitrile for more than 250 cycles. The excellent physical and electrochemical properties of the molecule suggest that **1i** would be an attractive anolyte for high-energy RFBs among the compounds studied herein. The theoretical capacity of the **1i**-based flow battery system (53.6 A hL^–1^) surpasses the capacity of unsubstituted azobenzene (46 A hL^–1^).^33^ Further experimental work is needed to evaluate the capacity fade and thus accessible capacity of potential **1i**-based batteries.

**Figure 3:**
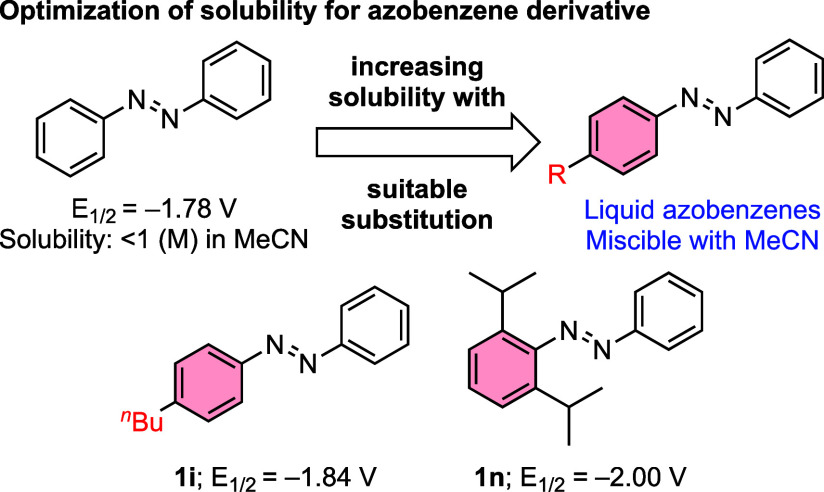
Development of liquid azobenzene toward achieving maximum concentration in NAORFBs.

### Electrokinetics

We examined the impact of the introduction of various substituents on the diffusion coefficient (*D*) and heterogeneous electron transfer kinetics (*k*_0_) of the azobenzene derivatives because the mass transport and electrokinetic behavior of charge carriers are crucial for achieving better-performing electrochemical cells with high power density. We define **1a** as a standard azobenzene derivative and investigated both *D* and *k*_0_ by collecting cyclic voltammetry (CV) data at scan rates from 25 to 500 mV/s. The peak current observed shows a linear relationship with the square root of the scan rate (Figure 4a), suggesting a diffusion-limited process. The diffusion coefficient was calculated according to the Randles–Ševcík equation to be *D* = 1.2 × 10^–5^ cm^2^·sec^–1^, which is comparable with unsubstituted azobenzene^33^ and other reported low-potential anolytes such as indolo­[2,3-*b*]­quinoxaline^30^ and *N*-methylphthalimide.^51^ Given the >60 mV peak-to-peak separation of the electrochemical events for the azobenzenes, the electron transfer rate constants obtained from the above analysis are underestimates.

**Figure 4:**
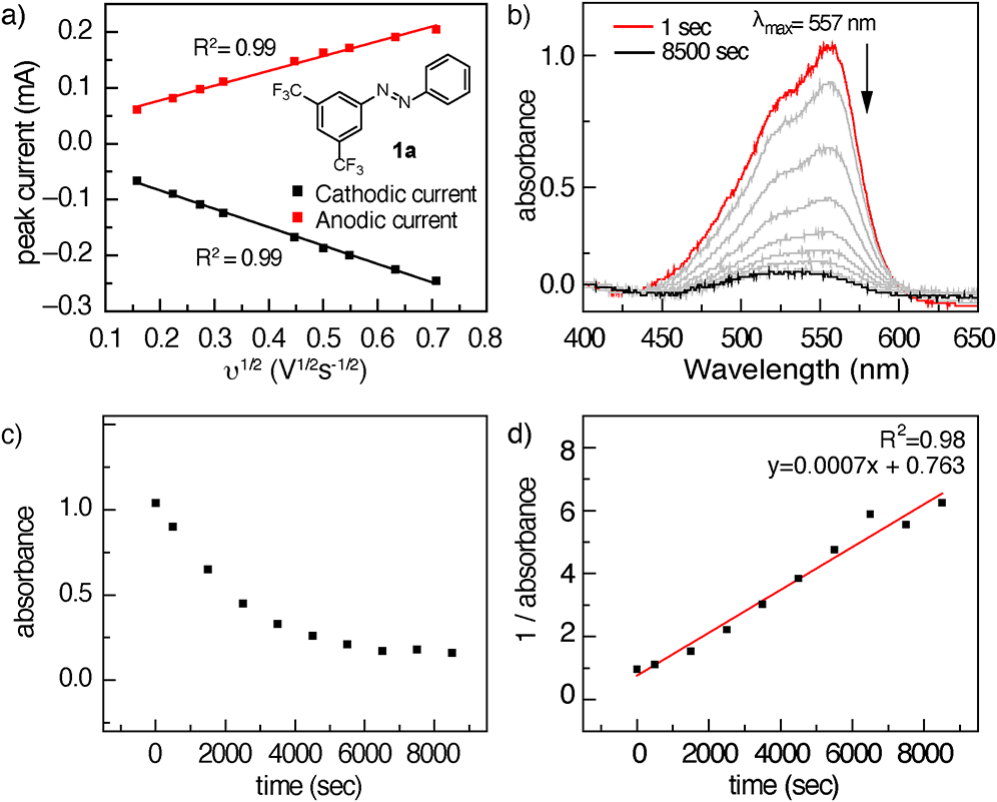
(a) Plot of anodic and cathodic peak current versus square root of the scan rate. (b) Decay of the monoreduced product after bulk electrolysis at a constant potential of −1.54 V vs. Fc^+^/Fc in MeCN. (c) Absorbance vs. time for the decay of **1a′**. (d) The linear correlation between inverse absorbance and time shows a second-order decay.

### Characterization and Decomposition of Monoreduced Azobenzenes

The stability and potential decomposition pathways of charged anolytes or catholytes are critical to understanding and minimizing the capacity fade of RFBs. To define the lifetime and decomposition mechanism of the charged state of the azobenzene, we first pursued spectroscopic characterization of the monoreduced species. These studies then enabled an examination of the kinetics for decomposition of this charged state. For this purpose, a 5 mM solution of **1a** was reduced in MeCN (containing 0.1 M TBAPF_6_) under an inert atmosphere with a constant potential of −1.54 V vs. Fc^+^/Fc until the color of the solution turned to wine red from orange; a total applied charge of 1 F/mol generated the monoreduced product, **1a′** (along with **1a** and **1a’’**, vide infra). The UV–vis spectrum of radical anion **1a′** displayed an absorbance centered at 557 nm (Figure 4b), which is a significant red shift of the corresponding feature in the absorption spectrum of noncharged **1a** (451 nm).^52,53^ We further confirmed that the observed spectral feature arises from **1a′** by independent synthesis: Treatment of **1a** with 1 equiv of Na metal gives rise to the same spectral feature centered at 557 nm that is characteristic of **1a′**. The distinct spectral features of **1a** and **1a′** enabled the study of time-dependent spectral evolution of **1a**′. Examination of the UV–vis spectrum of radical anion **1a** ^**′**^ as a function of time afforded the data pictured in Figure 4b. Plotting these data as absorbance vs. time shows a quadratic decay (Figure 4c), and plotting 1/absorbance vs. time shows a linear relationship with *R* ^2^ = 0.98, consistent with the second-order consumption of **1a** ^**′**^ (Figures 4d and S9). This second-order consumption is consistent with a disproportionation mechanism in which two equivalents of **1a′** react to generate an equivalent of dianion (**1a″**) and an equivalent of azobenzene (**1a**).^35,52,53^ Attempts to evaluate the decomposition kinetics of other azobenzene derivatives (**1**) were complicated by the modest solubilities (and thus solution opacity) of other radical anions (**1′**).

Disproportionation of monoanion **1a′** was further supported by spectral measurements made by 2-fold reduction of **1a** (generated by constant potential electrolysis at –2.15 V vs. Fc^+^/Fc). A signal at 366 nm in the UV–vis spectrum was observed, which was also found to slowly grow during electrolysis of **1a** at –1.54 V vs. Fc^+^/Fc (i.e., electrosynthesis of monoanion **1a**; Figure S10). The peak at 366 nm was stable and remained unchanged even after 24 h. This peak in the visible region is often characterized as a protonated azobenzene anion, formed by reaction between the azobenzene dianion and solvent proton (Scheme S1).^52^ The presence of the neutral azobenzene derivative was confirmed by ^1^H NMR spectroscopy (Figure S11). These observations indicate the slow generation of doubly reduced species upon disproportionation of the monoreduced radical anion (2­(**1a′**) → **1a** + **1a″**).

Next, we evaluated the water and oxygen sensitivity of both mono- and direduced products. In this case, we examined azobenzene derivative **1h**, which has two reversible reduction waves, unlike **1a**, which has only one reversible reduction wave. Compound **1h** has two reversible reduction waves at – 1.19 V and −1.81 V vs. Fc^+^/Fc with the cathodic to anodic peak ratio *i*_pa1_/*i*_pc1_ = 0.84 and *i*_pa2_/*i*_pc2_ = 0.69. While the ratio was largely unaffected with oxygen purging, a sharp change in the second reduction peak was observed with the addition of water (Figure S12). Indeed, upon the gradual addition of 50 equiv of water (relative to **1h**), the second reduction became completely irreversible, and the *i*_pa1_/*i*_pc1_ value of the first reduction decreased to 0.73. This suggests the fast decomposition or consumption of the doubly reduced species by water and the slower decomposition of the monoreduced species. However, the lower reactivity, and thus longer lifetime, of the azobenzene radical anion toward oxygen and water suggests the competency of azobenzene derivatives as low-potential anolytes with single-electron cycling furnishing minimum capacity fade after each cycle. Similar observations were made in galvanostatic charge–discharge cycling for **1h** (data collected in Figure S13). Significantly higher capacity was observed when only the one electron per azobenzene unit was cycled, but capacity degradation was observed for both one- and two-electron cycling experiments.

## Conclusion

To summarize our effort to define molecular design considerations for azobenzene anolytes for electrochemical energy storage, we synthesized a library of azobenzene derivatives with electron-donating and -withdrawing substituents as well as heteroaryl derivatives. This family displayed systematically variable reduction potential, redox inventory, melting point, and solubility. Whereas the azobenzene derivatives with alkyl substituents are liquid, and their low molecular weight can increase the active electrolyte concentration with a high charge/molecular weight ratio, only azobenzene derivatives with highly electronegative substituents stabilize the second reduction and eventually increase the number of electrons involved in reversible reduction. Moreover, electrochemical properties, such as mass transport and electrokinetics, remain unimpacted upon functionalization. Such properties, along with the long lifetime of monoreduced species, make modified azobenzene derivatives potential anolytes for high-performance NAORFBs. This study highlights the significance of derivatization for enhancing the properties of an anolyte as a route to produce a more efficient RFB, which may translate from azobenzenes to other electrolytes.

## Materials and Methods

All information detailing the materials and methods employed in this study is detailed in the accompanying Supporting Information.

### Synthesis of azobenzenes

Three procedures were used to prepare azobenzenes.

#### General Procedure B1: Coupling Anilines with Nitrosobenzene under Acidic Conditions

A 100 mL oven-dried round-bottom flask was charged with appropriate aniline (4.00 mmol, 1.00 equiv), nitrosobenzene (420 mg, 4.00 mmol, 1.00 equiv), acetic acid (AcOH, 10 mL), and ethanol (EtOH, 2.5 mL). The reaction mixture was heated at 40 °C for 12 h in an oil bath using a hot plate and then cooled to 23 °C. The mixture was diluted with CH_2_Cl_2_ (50 mL), washed with brine (3 × 25 mL), dried over anhydrous Na_2_SO_4_, filtered, and concentrated under reduced pressure. The crude reaction mixture was purified by column chromatography on silica gel.

#### General Procedure B2: Oxidative Coupling of Anilines

A 100 mL oven-dried round-bottom flask was charged with appropriate aniline (4.00 mmol, 1.00 equiv), MnO_2_ (3.47 g, 40.0 mmol, 10.0 equiv), and toluene (25 mL). The reaction mixture was heated to reflux for 12 h in an oil bath using a hot plate. The reaction mixture was cooled to 23 °C, filtered through Celite, dried, and purified by column chromatography on silica gel.

#### General Procedure B3: Coupling Anilines with Nitrosobenzene under Basic Conditions

A 100 mL oven-dried round-bottom flask was charged with appropriate aniline (11.6 mmol, 1.00 equiv) and benzene (1 mL). An aqueous solution of NaOH (556 mg, 13.9 mmol, 1.20 equiv in 6 mL H_2_O) was added slowly to the reaction mixture. Nitrosobenzene (1.24 g, 11.6 mmol, 1.00 equiv) was added over 15 min. The reaction mixture was heated to reflux for 10 min in an oil bath using a hot plate and then cooled to 23 °C. The organic layer was diluted with CH_2_Cl_2_ (50 mL), washed with brine (3 × 25 mL), dried over anhydrous Na_2_SO_4_, filtered, and concentrated under reduced pressure. The crude reaction mixture was purified by column chromatography on a silica gel.

## Supplementary Material



## Data Availability

The data underlying this study are available in the published article and its Supporting Information.

## Notes

Experimental methods, optimization data, spectral data, and experimental spectra (PDF)

## References

[1] Nayak P. K., Mahesh S., Snaith H. J., Cahen D. (2019). Photovoltaic Solar
Cell Technologies: Analysing the State of the Art. Nat. Rev. Mater..

[2] Egbert G., Ray R. (2000). Significant Dissipation
of Tidal Energy in the Deep Ocean Inferred
from Satellite Altimeter Data. Nature.

[3] Aron N. S. M., Khoo K. S., Chew K. W., Show P. L., Chen W.-H., Nguyen T. H. P. (2020). Sustainability of The Four Generations of Biofuels
– A Review. Int. J. Energy Res..

[4] Zeng Y. K., Zhao T. S., An L., Zhou X. L., Wei L. (2015). A Comparative
Study of All-Vanadium and Iron-Chromium Redox Flow Batteries for Large-Scale
Energy Storage. J. Power Sources.

[5] Tian Y., Zeng G., Rutt A., Shi T., Kim H., Wang J., Koettgen J., Sun Y., Ouyang B., Chen T., Lun Z., Rong Z., Persson K., Ceder G. (2021). Promises and Challenges of Next-Generation
“Beyond Li-ion”
Batteries for Electric Vehicles and Grid Decarbonization. Chem. Rev..

[6] Samaroo S., Hengesbach C., Bruggeman C., Carducci N. G. G., Mtemeri L., Staples R. J., Guarr T., Hickey D. P. (2023). C–H···π
Interactions Disrupt Electrostatic Interactions between Non-Aqueous
Electrolytes to Increase Solubility. Nat. Chem..

[7] Attanayake N. H., Kowalski J. A., Greco K. V., Casselman M. D., Milshtein J. D., Chapman S. J., Parkin S. R., Brushett F. R., Odom S. A. (2019). Tailoring Two-Electron-Donating Phenothiazines
to Enable
High-Concentration Redox Electrolytes for Use in Nonaqueous Redox
Flow Batteries. Chem. Mater..

[8] Kowalski J. A., Casselman M. D., Kaur A. P., Milshtein J. D., Elliott C. F., Modekrutti S., Attanayake N. H., Zhang N., Parkin S. R., Risko C., Brushett F. R., Odom S. A. (2017). A Stable Two-Electron-Donating Phenothiazine for Application
in Nonaqueous Redox Flow Batteries. J. Mater.
Chem. A.

[9] Escamilla M., Zuleta E. C., Davis H. K., Johnson J., Pentzer E., Zawodzinski T. (2024). Synthesis
of Alkoxy-TEMPO Aminoxyl Radicals and Electrochemical
Characterization in Acetonitrile for Energy Storage Applications. J. Electrochem. Soc..

[10] Zhang L., Feng R., Wang W., Yu G. (2022). Emerging Chemistries
and Molecular Designs for Flow Batteries. Nat.
Rev. Chem..

[11] Luo J., Hu B., Hu M., Zhao Y., Liu T. L. (2019). Status and Prospects
of Organic Redox Flow Batteries toward Sustainable Energy Storage. ACS Energy Lett..

[12] Pan M., Gao L., Liang J., Zhang P., Lu S., Lu Y., Ma J., Jin Z. (2022). Reversible Redox Chemistry in Pyrrolidinium-Based TEMPO
Radical and Extended Viologen for High-Voltage and Long-Life Aqueous
Redox Flow Batteries. Adv. Energy Mater..

[13] Hu M., Wu W., Luo J., Liu T. L. (2022). Desymmetrization of Viologen Anolytes
Empowering Energy Dense, Ultra Stable Flow Batteries toward Long-Duration
Energy Storage. Adv. Energy Mater..

[14] Li H., Fan H., Hu B., Hu L., Chang G., Song J. (2021). Spatial Structure
Regulation: A Rod-Shaped Viologen Enables Long Lifetime in Aqueous
Redox Flow Batteries. Angew. Chem., Int. Ed..

[15] Gao M., Salla M., Song Y., Wang Q. (2022). High-Power Near-Neutral
Aqueous All Organic Redox Flow Battery Enabled with a Pair of Anionic
Redox Species. Angew. Chem., Int. Ed..

[16] Lv X.-L., Sullivan P., Fu H.-C., Hu X., Liu H., Jin S., Li W., Feng D. (2022). Dextrosil-Viologen:
A Robust and
Sustainable Anolyte for Aqueous Organic Redox Flow Batteries. ACS Energy Lett..

[17] Hu B., Hu M., Luo J., Liu T. L. (2022). A Stable, Low Permeable TEMPO Catholyte
for Aqueous Total Organic Redox Flow Batteries. Adv. Energy Mater..

[18] Fan H., Liu K., Zhang X., Di Y., Liu P., Li J., Hu B., Li H., Ravivarma M., Song J. (2024). Spatial Structure Regulation
Towards Armor-Clad Five-Membered Pyrroline Nitroxides Catholyte for
Long-Life Aqueous Organic Redox Flow Batteries. eScience.

[19] Fan H., Wu W., Ravivarma M., Li H., Hu B., Lei J., Feng Y., Sun X., Song J., Liu T. L. (2022). Mitigating
Ring-Opening to Develop Stable TEMPO Catholytes for pH-Neutral All-Organic
Redox Flow Batteries. Adv. Funct. Mater..

[20] Noack J., Roznyatovskaya N., Herr T., Fischer P. (2015). The Chemistry of Redox-Flow
Batteries. Angew. Chem., Int. Ed..

[21] Zhao Y., Ding Y., Li Y., Peng L., Byon H. R., Goodenough J. B., Yu G. A. (2015). A Chemistry and
Material Perspective
on Lithium Redox Flow Batteries Towards High-Density Electrical Energy
Storage. Chem. Soc. Rev..

[22] Huang Y., Gu S., Yan Y., Li S. F. Y. (2015). Nonaqueous Redox-Flow Batteries:
Features, Challenges, and Prospects. Curr. Opin.
Chem. Eng..

[23] Li M., Rhodes Z., Cabrera-Pardo J. R., Minteer S. D. (2020). Recent Advancements
in Rational Design of Non-Aqueous Organic Redox Flow Batteries. Sustainable Energy Fuels.

[24] Bockman T. M., Kochi J. K. (1990). Isolation and Oxidation-Reduction of Methylviologen
Cation Radicals. Novel Disproportionation in Charge-Transfer Salts
by X-Ray Crystallography. J. Org. Chem..

[25] Assary R. S., Zhang L., Huang J., Curtiss L. A. (2016). Molecular
Level
Understanding of the Factors Affecting the Stability of Dimethoxy
Benzene Catholyte Candidates from First-Principles Investigations. J. Phys. Chem. C.

[26] Wei X., Xu W., Huang J., Zhang L., Walter E., Lawrence C., Vijayakumar M., Henderson W. A., Liu T., Cosimbescu L., Li B., Sprenkle V., Wang W. (2015). Radical Compatibility with Nonaqueous
Electrolytes and Its Impact on an All-Organic Redox Flow Battery. Angew. Chem., Int. Ed..

[27] Peltier C. R., Rhodes Z., Macbeth A. J., Milam A., Carroll E., Coates G. W., Minteer S. D. (2022). Suppressing Crossover in Nonaqueous
Redox Flow Batteries with Polyethylene-Based Anion-Exchange Membranes. ACS Energy Lett..

[28] Perry M. L., Saraidaridis J. D., Darling R. M. (2020). Crossover Mitigation Strategies for
Redox-Flow Batteries. Curr. Opin. Electrochem..

[29] Arévalo-Cid P., Dias P., Mendes A., Azevedo J. (2021). Redox Flow Batteries:
A New Frontier on Energy Storage. Sustainable
Energy Fuels.

[30] Zhang W., Walser-Kuntz R., Tracy J. S., Schramm T. K., Shee J., Head-Gordon M., Chen G., Helms B. A., Sanford M. S., Toste F. D. (2023). Indolo­[2,3-b]­quinoxaline
as a Low Reduction Potential
and High Stability Anolyte Scaffold for Nonaqueous Redox Flow Batteries. J. Am. Chem. Soc..

[31] Yan Y., Zhang L., Walser-Kuntz R., Vogt D. B., Sigman M. S., Yu G., Sanford M. S. (2022). Benzotriazoles as Low-Potential Anolytes for Non-aqueous
Redox Flow Batteries. Chem. Mater..

[32] Sevov C. S., Hickey D. P., Cook M. E., Robinson S. G., Barnett S., Minteer S. D., Sigman M. S., Sanford M. S. (2017). Physical Organic
Approach to Persistent, Cyclable, Low-Potential Electrolytes for Flow
Battery Applications. J. Am. Chem. Soc..

[33] Zhang L., Qian Y., Feng R., Ding Y., Zu X., Zhang C., Guo X., Wang W., Yu G. (2020). Reversible
Redox Chemistry in Azobenzene-Based Organic Molecules for High-Capacity
and Long-Life Nonaqueous Redox Flow Batteries. Nat. Commun..

[34] Zu X., Zhang L., Qian Y., Zhang C., Yu G. (2020). Molecular
Engineering of Azobenzene-Based Anolytes Towards High-Capacity Aqueous
Redox Flow Batteries. Angew. Chem., Int. Ed..

[35] Wang X., Chai J., Lashgari A., Jiang J. J. (2021). Azobenzene-Based
Low-Potential Anolyte for Nonaqueous Organic Redox Flow Batteries. ChemElectroChem..

[36] Shimizu T., Tanifuji N., Yoshikawa H. (2022). Azo Compounds
as Active Materials
of Energy Storage Systems. Angew. Chem., Int.
Ed..

[37] Xu D., Zhang C., Zhen Y., Zhao Y., Li Y. (2021). A High-Rate
Nonaqueous Organic Redox Flow Battery. J. Power
Sources.

[38] Chen Y., Dai H. C., Fan K., Zhang G., Tang M., Gao Y., Zhang C., Guan L., Mao M., Liu H., Zhai T., Wang C. (2023). A Recyclable and Scalable High-Capacity
Organic Battery. Angew. Chem., Int. Ed..

[39] Du D., Chen Y., Zhang H., Zhao J., Jin L., Ji W., Huang H., Pang S. (2024). High-Performance Azo Cathodes Enabled
by N-Heteroatomic Substitution for Zinc Batteries with a Self-Charging
Capability. Angew. Chem., Int. Ed..

[40] Han B., Jiao H., Chen R., Zhang Y., Wang J. (2023). Chemoselective
Reduction of Imines and Azobenzenes Catalyzed by Silver N-Heterocyclic
Carbene Complexes. Org. Chem. Front..

[41] Das U. K., Kar S., Ben-David Y., Diskin-Posner Y., Milstein D. (2021). Manganese Catalyzed
Hydrogenation of Azo (N=N) Bonds to Amines. Adv. Synth. Catal..

[42] Balam-Villarreal J. A., López-Mayorga B. J., Gallardo-Rosas D., Toscano R. A., Carreón-Castro M. P., Basiuk V. A., Cortés-Guzmán F., López-Cortés J. G., Ortega-Alfaro M. C. (2020). π-Extended
Push–Pull Azo-Pyrrole Photoswitches:
Synthesis, Solvatochromism and Optical Band Gaps. Org. Biomol. Chem..

[43] Weston C. E., Richardson R. D., Haycock P. R., White A. J. P., Fuchter M. J. (2014). Arylazopyrazoles:
Azoheteroarene Photoswitches Offering Quantitative Isomerization and
Long Thermal Half-Lives. J. Am. Chem. Soc..

[44] Dougan S. J., Melchart M., Habtemariam A., Parsons S., Sadler P. J. (2006). Phenylazo-pyridine
and Phenylazo-pyrazole Chlorido Ruthenium­(II) Arene Complexes: Arene
Loss, Aquation, and Cancer Cell Cytotoxicity. Inorg. Chem..

[45] Zhu Q., Costentin C., Stubbe J., Nocera D. G. (2023). Disulfide readical
anion as a super-reductant in biology and photoredox chemistry. Chem. Sci..

[46] Frey B. L., Thai P., Patel L., Powers D. C. (2023). Structure-Activity
Relationships in Hypervalent Iodine Electrocatalysis. Synthesis.

[47] Markiewicz R., Klimaszyk A., Jarek M., Taube M., Florczak P., Kempka M., Fojud Z., Jurga S. (2021). Influence of Alkyl
Chain Length on Thermal Properties, Structure, and Self-Diffusion
Coefficients of Alkyltriethylammonium-Based Ionic Liquids. Int. J. Mol. Sci..

[48] Wight C. D., Xiao Q., Wagner H. R., Hernandez E. A., Martin E., Lynch V. M., Iverson B. L. (2022). Influence
of the
Alkyl Side Chain Length on the Assembly and Thermochromic Solid-State
Properties in Symmetric and Asymmetric Monoalkoxynaphthalene–Naphthalimide
Donor–Acceptor Dyads. Cryst. Growth Des..

[49] Pinal R. (2004). Effect of
Molecular Symmetry on Melting Temperature and Solubility. Org. Biomol. Chem..

[50] Boobier S., Hose D. R. J., Blacker A. J., Nguyen B. N. (2020). Machine Learning
with Physicochemical Relationships: Solubility Prediction in Organic
Solvents and Water. Nat. Commun..

[51] Wei X., Duan W., Huang J., Zhang L., Li B., Reed D., Xu W., Sprenkle V., Wang W. (2016). A High-Current,
Stable Nonaqueous Organic Redox Flow Battery. ACS Energy Lett..

[52] Boto K. G., Thomas F. G. (1973). The Polarography
of Some Substituted Azobenzenes in
Acetonitrile. II. Identification of Reduction Products. Aust. J. Chem..

[53] Glotz G., Knaipp K., Maier M. S., Hüll K., Novak A., Kelterer A.-M., Griebenow T., Herges R., Trauner D., Gescheidt G. (2023). To Isomerize
or not to Isomerize? E/Z Isomers of Cyclic Azobenzene Derivatives
and Their Reactivity Upon One Electron Reduction. Chem.–Eur. J..

