# Supplementary material for: Molecular Design Considerations for Azobenzene Anolytes
Source: ACS Omega. 2025 Jun 12;10(24):26199–206. doi: 10.1021/acsomega.5c05073 (PMC12199068; doi:10.1021/acsomega.5c05073)
Supplement: Supplementary file 1 [file ao5c05073_si_001.pdf]

Supporting Information

Molecular Design Considerations for Azobenzene Anolytes

Ananya Banik,[a] Uddalak Sengupta,[a] Haley Hughes,[a] Palani Sabhapathy,[b] Burcu Gurkan,[b,*] Emily B. Pentzer,[a,*] David C. Powers[a,*]

[a] Department of Chemistry, Texas A&M University, College Station, Texas, United States

[b] Case Western Reserve University, Department of Chemical and Biomolecular Engineering, Cleveland, Ohio 44106, United States

Email: beg23@case.edu, emilypentzer@tamu.edu, powers@chem.tamu.edu

Table of Contents

A. General Considerations
  A.1 Materials S3
  A.2 Characterization Details S3
  A.3 Solubility Measurements S4
  A.4 Safety Statement S4
B. Synthesis of Azobenzene Derivatives S5
C. Supporting Data S13
D. Electrochemical and Spectroscopic Characterization S27
E. NMR Spectra of New Compounds S46
F. References S52

A. General Considerations

A.1 Materials Tetrabutylammonium hexafluorophosphate ($TBAPF_6$) was purchased from Oakwood Chemicals and recrystallized from boiling ethanol. All other chemicals and solvents were obtained as ACS reagent grade and used as received. 2,6-Diisopropylaniline, 4-nitroaniline, 4′-aminoacetophenone, *N*-methylpyrrole, 2-aminopyridine, silica gel (0.060–0.200 mm, 60A for column chromatography), methanol, hexanes, ethyl acetate, dichloromethane, and acetic acid were obtained from Sigma Aldrich. 3,5-Bis(trifluoromethyl)aniline, 4-*tert*-butylaniline, 4-aminobenzonitrile, 4-aminobiphenyl, 4-aminopyridine, and *N*,*N*-dimethylbenzene-1,4-diamine were purchased from Oakwood Chemicals. *p*-Anisidine and ferrocene were obtained from Alfa Aesar. 4-(Trifluoromethyl)aniline, 4-*n*-butylaniline, (4-aminophenyl)(phenyl)methanone, 1-methyl-1H-pyrazol-4-amine, isoxazol-3-amine, benzo[d]thiazol-2-amine, and nitrosobenzene were purchased from Ambeed. Hydrochloric acid, acetonitrile, and ethanol (200 proof) were obtained from Fischer Scientific. NMR solvents were purchased from Cambridge Isotope Laboratories and were used as received. Experiments were carried out under ambient atmosphere unless otherwise noted.

A.2 Characterization Details ^{1}H spectra were recorded on an Acsend™ 400 NMR (Bruker) and were referenced against the residual proteo solvent signal: $CDCl_3$ (7.26 ppm, ^{1}H; 77.16 ppm, ^{13}C) acetonitrile-d_3 (1.94 ppm, ^{1}H, 1.32 ppm, ^{13}C).[1] ^{1}H NMR data are reported as follows: chemical shift (δ, ppm), (multiplicity: s (singlet), d (doublet), t (triplet), m (multiplet), br (broad), integration). Mass spectrometry data was recorded on either Orbitrap Fusion™ Tribrid™ Mass Spectrometer or Q Exactive™ Focus Hybrid Quadrupole-Orbitrap™ Mass Spectrometer from ThermoFisher Scientific. Cyclic voltammetry (CV) was carried out at 23 °C using CH Instruments Electrochemical Analyzer (Model CHI620A) in a three-electrode cell using glassy carbon working electrode, Pt counter electrode, and Ag-reference electrode obtained from IKA. All CV experiments were carried out in 10-mL Electrasyn glass vial with freshly polished electrodes using CH instruments electrode polishing kit. Reference electrode was prepared using 0.1 M solution of $TBAPF_6$ in acetonitrile with 1.0 mM $AgNO_3$. The solvent was deoxygenated by purging nitrogen into the electrolyte solution for 5 minutes and CV was measured under constant flow of nitrogen gas on the head space. For all CV experiments, the initial potential was set to 0 V and the reductive scan was performed first. Bulk electrolysis experiments were carried out using CH Instruments Electrochemical Analyzer (Model CHI620A) in a three-electrode cell using a glassy carbon/Pt working electrode, Pt counter electrode, and Ag-reference electrode obtained from IKA. All electrochemical experiments were carried out in 10-mL Electrasyn glass vial with freshly polished electrodes. Reference electrodes were prepared using 0.1 M solution of $TBAPF_6$ in acetonitrile with 1.0 mM $AgNO_3$. UV-vis spectra were recorded at 293 K in quartz cuvettes on an Ocean Optics Flame-S miniature spectrometer with DH-mini UV-vis NIR light source (200–900 nm) and were blanked against the appropriate solvent. EPR spectra were recorded at 8 K on a Bruker ELEXSYS E500 X-band

spectrometer equipped with a ER4102ST resonator using the following experimental parameters: 1024 points with 20 mW microwave power, 0.01 mT modulation amplitude, 100 kHz modulation frequency, and a sweep range of 5 mT.

A.3 Solubility Measurements Each azobenzene derivative was added to 0.5 mL acetonitrile slowly with occasional sonication until a solid persisted. The suspension was filtered through a PTFE syringe filter to remove the solids, and the saturated solution of the azobenzene derivative was collected. Aliquots (2 × 100 μL) of the saturated solution was transferred in two different vials, and dried *in vacuo*. The weight of the residue was then measured and the solubility was calculated.

A.4 Galvanostatic Cycling Charge/discharge measurements with a custom glass H-cell were carried out in an Ar-filled glovebox using a BioLogic VSP galvanostat. The working and counter electrodes consisted of a reticulated vitreous carbon (RVC) electrode (100 ppi), while the separator was Daramic 175. An Ag/Ag^+ reference electrode was used on the working side of the H-cell. The electrolyte contained 5 mM active species and 0.5 M $TEABF_4$ in ACN. Both chambers of the H-cell were loaded with 6 mL of the electrolyte solution, and their contents were stirred continuously. For the initial charge, 1 C-rate was applied with a potential cutoff of –2.20 V *vs.* Ag/Ag^+. After the initial charge, the solution on the counter electrode side was exchanged for a fresh solution to enable symmetrical one-electron cycling. The working side was charged at 5 mA (approximately 5 C). Voltage cutoffs were set at 0.3 V higher than $E_{1/2}$ as the upper limit and −0.3 V lower than $E_{1/2}$ as the lower limit for both one-electron and two-electron processes for **1h**.

A.5 Safety Statement No significant hazard or safety risk was encountered during this work. Standard laboratory safety equipment and procedures should be employed at all times.

B. Synthesis of Azobenzene Derivatives

General Procedure B1

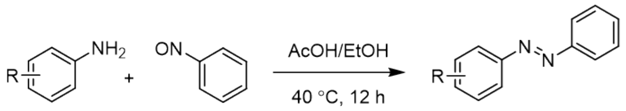

A 100-mL oven-dried round bottom flask was charged with appropriate aniline (4.00 mmol, 1.00 equiv), nitrosobenzene (420 mg, 4.00 mmol, 1.00 equiv), acetic acid (AcOH, 10 mL), and ethanol (EtOH, 2.5 mL). The reaction mixture was heated at 40 °C for 12 h in an oil bath using a hot plate and then cooled to 23 °C. The mixture was diluted with CH_2Cl_2 (50 mL), washed with brine (3 × 25 mL), dried over anhydrous Na_2SO_4, filtered, and concentrated under reduced pressure. The crude reaction mixture was purified by column chromatography on silica gel.

General Procedure B2

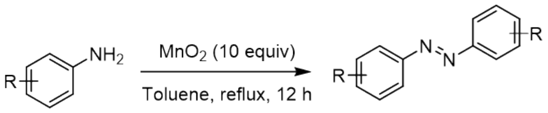

A 100-mL oven-dried round bottom flask was charged with appropriate aniline (4.00 mmol, 1.00 equiv), MnO_2 (3.47 g, 40.0 mmol, 10.0 equiv), and toluene (25 mL). The reaction mixture was heated to reflux for 12 h in an oil bath using a hot plate. The reaction mixture was cooled to 23 °C, filtered through Celite, dried, and purified by column chromatography on silica gel.

General Procedure B3

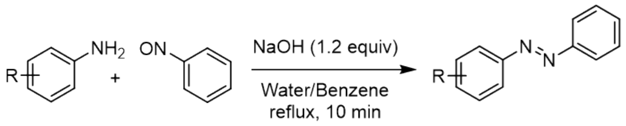

A 100-mL oven-dried round bottom flask was charged with appropriate aniline (11.6 mmol, 1.00 equiv) and benzene (1 mL). An aqueous solution of NaOH (556 mg, 13.9 mmol, 1.20 equiv in 6 mL H_2O) was added slowly to the reaction mixture. Nitrosobenzene (1.24 g, 11.6 mmol, 1.00 equiv) was added over 15 min. The reaction mixture was heated to reflux for 10 min in an oil bath using a hot plate and then cooled to 23 °C. The organic layer was diluted with CH_2Cl_2 (50 mL), washed with brine (3 × 25 mL), dried over anhydrous Na_2SO_4, filtered, and concentrated under reduced pressure. The crude reaction mixture was purified by column chromatography on silica gel.

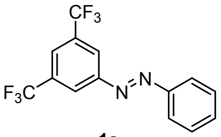

1a

(E)-1-(3,5-bis(trifluoromethyl)phenyl)-2-phenyldiazene (1a) Azobenzene derivative **1a** was synthesized according to general procedure B1 from 3,5-bis(trifluoromethyl)aniline and nitrosobenzene, purified by silica gel column chromatography with a hexanes/ethyl acetate (9/1) eluent, and obtained as orange crystalline solid (1.04 g, 82% yield), m.p. 128 °C. ^{1}H NMR ($CDCl_3$, 400 MHz, 298 K): δ 8.37 (s, 2H), 7.99–7.97 (m, 3H), 7.57–7.55 (m, 3H) ppm. The obtained spectral data are well-matched to those in the literature.[4]

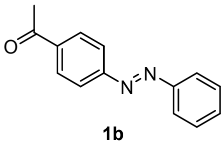

1b

(E)-1-(4-(phenyldiazenyl)phenyl)ethan-1-one (1b) Azobenzene derivative **1b** was synthesized according to general procedure B1 from 1-(4-aminophenyl)ethan-1-one and nitrosobenzene, purified by silica gel column chromatography with a hexanes/ethyl acetate (9/1) eluent, and obtained as orange crystalline solid (690 mg, 77% yield), m.p. 115 °C. ^{1}H NMR ($CDCl_3$, 400 MHz, 298 K): δ 7.93 (td, 2H, J = 8.8 Hz, 2.0 Hz), 7.81 (tt, 3H, J = 8.8 Hz, 1.6 Hz,), 7.38–7.36 (m, 3H), 2.48 (s, 3H) ppm. The obtained spectral data are well-matched to those in the literature.[5]

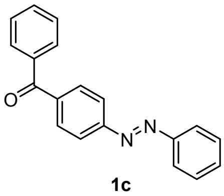

1c

(E)-phenyl(4-(phenyldiazenyl)phenyl)methanoneone (1c) Azobenzene derivative **1c** was synthesized according to general procedure B1 from (4-aminophenyl)(phenyl)methanone and nitrosobenzene, purified by silica gel column chromatography with a hexanes/ethyl acetate (9/1) eluent, and obtained as orange crystalline solid (847 mg, 74% yield), m.p. 110 °C. ^{1}H NMR ($CDCl_3$, 400 MHz, 298 K): δ 7.88-7.81 (m, 6H), 7.70 (d, 2H, J = 7.2 Hz), 7.47 (t, 1H, J = 7.2 Hz), 7.42–7.35 (m, 5H) ppm. $^{13}C\{^{1}H\}$ NMR ($CDCl_3$, 101 MHz, 298 K): δ 196.0, 154.6, 152.6, 139.2, 137.4, 132.7, 131.8, 131.1, 130.1, 129.2, 128.4, 123.2, 122.7 ppm. HRMS-ESI$^+$ (m/z): $[M+H]^+$ calcd. For $C_{19}H_{15}N_2O^+$, 287.1179; found, 287.1172.

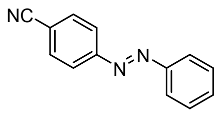

1d

(E)-4-(phenyldiazenyl)benzonitrile (1d) Azobenzene derivative **1d** was synthesized according to general procedure B1 from 4-aminobenzonitrile and nitrosobenzene, purified by silica gel column chromatography with a hexanes/ethyl acetate (9/1) eluent, and obtained as yellow crystalline solid (630 mg, 76% yield), m.p. 122 °C. ^{1}H NMR ($CDCl_3$, 400 MHz, 298 K): δ 7.99 (td, 2H, *J* = 8.8 Hz, 2 Hz), 7.97–7.96 (m, 1H), 7.94 (d, 1H, *J* = 2.4 Hz), 7.82 (d, 2H, *J* = 8.4 Hz), 7.56–7.50 (m, 3H) ppm. The obtained spectral data are well-matched to those in the literature.[6]

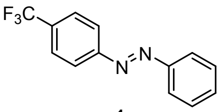

1e

(E)-1-phenyl-2-(4-(trifluoromethyl)phenyl)diazene (1e) Azobenzene derivative **1d** was synthesized according to general procedure B1 from 4-(trifluoromethyl)aniline and nitrosobenzene, purified by silica gel column chromatography with a hexanes/ethyl acetate (9/1) eluent, and obtained as orange crystalline solid (790 mg, 79% yield), m.p. 98 °C. ^{1}H NMR ($CDCl_3$, 400 MHz, 298 K): δ 7.99–7.95 (m, 4H), 7.76 (d, 2H, *J* = 8.4 Hz), 7.53–7.51 (m, 3H), 7.82 (d, 2H, *J* = 8.4 Hz), 7.56–7.50 (m, 3H) ppm. The obtained spectral data are well-matched to those in the literature.[7]

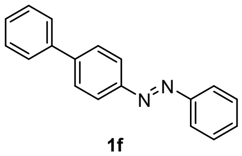

1f

(E)-1-([1,1'-biphenyl]-4-yl)-2-phenyldiazene (1f) Azobenzene derivative **1f** was synthesized according to general procedure B1 from [1,1'-biphenyl]-4-amine and nitrosobenzene, purified by silica gel column chromatography with a hexanes/ethyl acetate (9/1) eluent, and obtained as orange crystalline solid (826 mg, 80% yield), m.p. 155 °C. ^{1}H NMR ($CDCl_3$, 400 MHz, 298 K): δ 7.98 (d, 2H, *J* = 8.0 Hz), 7.95 (d, 2H, *J* = 4.0 Hz), 7.75 (d, 2H, *J* = 4.0 Hz), 7.64 (d, 2H, *J* = 8.0 Hz), 7.53–7.41 (m, 5H), 7.39–7.36 (m, 1H) ppm. The obtained spectral data are well-matched to those in the literature.[8]

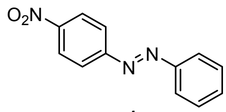

1g

(E)-1-(4-nitrophenyl)-2-phenyldiazene (1g) Azobenzene derivative **1g** was synthesized according to general procedure B1 from 4-nitroaniline and nitrosobenzene, purified by silica gel column chromatography with a hexanes/ethyl acetate (8/2) eluent, and obtained as red crystalline solid (608 mg, 67% yield), m.p. 133 °C. ^{1}H NMR ($CDCl_3$, 400 MHz, 298 K): δ 8.29 (d, 2H, J = 9.2 Hz), 7.94 (d, 2H, J = 8.8 Hz), 7.89–7.87 (m, 2H), 7.48–7.46 (m, 3H) ppm. The obtained spectral data are well-matched to those in the literature.[9]

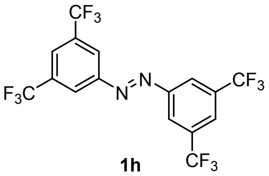

1h

(E)-1,2-bis(3,5-bis(trifluoromethyl)phenyl)diazene (1h) Azobenzene derivative **1g** was synthesized according to general procedure B2 from 3,5-bis(trifluoromethyl)aniline, purified by silica gel column chromatography with a hexanes/ethyl acetate (9/1) eluent, and obtained as red crystalline solid (708 mg, 78% yield), m.p. 128 °C. ^{1}H NMR ($CDCl_3$, 400 MHz, 298 K): δ 8.45 (s, 4H), 8.06 (s, 2H) ppm. The obtained spectral data are well-matched to those in the literature.[10]

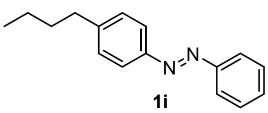

1i

(E)-1-(4-butylphenyl)-2-phenyldiazene (1i) Azobenzene derivative **1g** was synthesized according to general procedure B1 from 4-*n*-butyl aniline and nitrosobenzene, purified by silica gel column chromatography with a hexanes/ethyl acetate (9/1) eluent, and obtained as a red oil (781 mg, 82% yield), m.p. <23 °C. ^{1}H NMR ($CDCl_3$, 400 MHz, 298 K): δ 7.79 (d, 2H, J = 7.2 Hz), 7.74 (d, 2H, J = 8.4 Hz), 7.37 (t, 2H, J = 7.2 Hz), 7.33–7.29 (m, 1H), 7.18 (d, 2H, J = 8.4 Hz), 2.55 (t, 2H, J = 7.6 Hz), 1.55–1.48 (m, 2H), 1.25 (h, 2H, J = 7.6 Hz), 0.08 (t, 3H, J = 7.6 Hz) ppm. ^{13}C{^{1}H} NMR ($CDCl_3$, 101 MHz, 298 K): δ 152.9, 151.1, 146.6, 130.8, 129.2, 129.1, 123.0, 122.8, 35.7, 33.5, 22.5, 14.1 ppm. HRMS-ESI^{+} (m/z): $[M+H]^{+}$ calcd. For $C_{16}H_{19}N_2^{+}$, 239.1543; found, 239.1537.

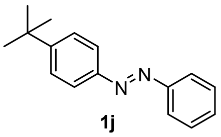

1j

(E)-1-(4-(tert-butyl)phenyl)-2-phenyldiazene (1j) Azobenzene derivative **1j** was synthesized according to general procedure B1 from 4-(*tert*-butyl)aniline and nitrosobenzene, purified by silica gel column chromatography with a hexanes/ethyl acetate (9/1) eluent, and obtained as orange crystalline solid (733 mg, 77% yield), m.p. <54 °C. ^{1}H NMR ($CDCl_3$, 400 MHz, 298 K): δ 8.07–8.00 (m, 4H), 7.65–7.57 (m, 4H), 7.53 (tt, 1H, *J* = 7.2 Hz, 1.6 Hz), 1.47 (s, 9H) ppm. The obtained spectral data are well-matched to those in the literature.[11]

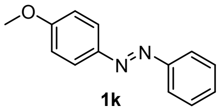

1k

(E)-1-(4-methoxyphenyl)-2-phenyldiazene (1k) Azobenzene derivative **1j** was synthesized according to general procedure B1 from 4-methoxyaniline and nitrosobenzene, purified by silica gel column chromatography with a hexanes/ethyl acetate (7/3) eluent, and obtained as yellow solid (611 mg, 72% yield), m.p. 58 °C. ^{1}H NMR ($CDCl_3$, 400 MHz, 298 K): δ 7.95–7.88 (m, 4H), 7.51 (t, 2H, *J* = 6.8 Hz), 7.44 (tt, 1H, *J* = 7.6 Hz ,1.2 Hz), 7.02 (d, 2H, *J* = 8.8 Hz), 3.89 (s, 3H) ppm. The obtained spectral data are well-matched to those in the literature.[12]

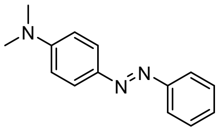

1l

(E)-N,N-dimethyl-4-(phenyldiazenyl)aniline (1l) Azobenzene derivative **1l** was synthesized according to general procedure B1 from *N1*,*N1*-dimethylbenzene-1,4-diamine and nitrosobenzene, purified by silica gel column chromatography with a hexanes/ethyl acetate (8/2) eluent, and obtained as orange solid (613 mg, 68% yield), m.p. 119 °C. ^{1}H NMR ($CDCl_3$, 400 MHz, 298 K): δ 7.91–7.84 (m, 4H), 7.48 (tt, 2H, *J* = 7.2 Hz, 1.6 Hz), 7.38 (tt, 1H, *J* = 7.6 Hz, 1.6 Hz), 6.77 (d, 2H, *J* = 8.8 Hz), 3.08 (s, 6H) ppm. The obtained spectral data are well-matched to those in the literature.[13]

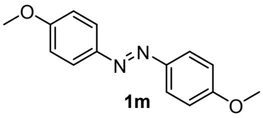

1m

(E)-1,2-bis(4-methoxyphenyl)diazene (1m) Azobenzene derivative **1m** was synthesized according to general procedure B2 from 4-methoxyaniline, purified by silica gel column chromatography with a hexanes/ethyl acetate (8/2) eluent, and obtained as yellow solid (329 mg, 68% yield), m.p. 165 °C. ^{1}H NMR ($CDCl_3$, 400 MHz, 298 K): δ 7.89 (d, 4H, J = 8.8 Hz), 7.00 (d, 4H, J = 9.2 Hz), 3.88 (s, 6H) ppm. The obtained spectral data are well-matched to those in the literature.[14]

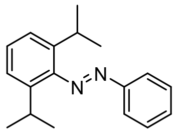

1n

(E)-1-(2,6-diisopropylphenyl)-2-phenyldiazene (1n) Azobenzene derivative **1n** was synthesized according to general procedure B1 from 2,6-diisopropylaniline and nitrosobenzene, purified by silica gel column chromatography with a hexanes/ethyl acetate (8/2) eluent, and obtained as orange oil (682 mg, 64% yield), m.p. <23 °C. ^{1}H NMR ($CDCl_3$, 400 MHz, 298 K): δ 7.96–7.92 (m, 2H), 7.58–7.54 (m, 3H), 7.30–7.26 (m, 3H), 3.11–3.05 (m, 2H), 1.23–1.20 (m, 12H) ppm. The obtained spectral data are well-matched to those in the literature.[15]

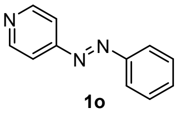

1o

(E)-4-(phenyldiazenyl)pyridine (1o) Azobenzene derivative **1o** was synthesized according to general procedure B3 from 4-aminopyridine and nitrosobenzene, purified by silica gel column chromatography with a hexanes/ethyl acetate (8/2) eluent, and obtained as orange crystalline solid (1.63 g, 79% yield), m.p. 101 °C. ^{1}H NMR ($CDCl_3$, 400 MHz, 298 K): δ 8.81 (dd, 2H, J = 4.4 Hz, 1.6 Hz), 7.98–7.95 (m, 2H), 7.71 (dd, 2H, J = 4.4 Hz, 1.6 Hz), 7.57–7.54 (m, 3H) ppm. The obtained spectral data are well-matched to those in the literature.[16]

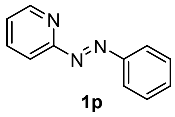

1p

(E)-2-(phenyldiazenyl)pyridine (1p) Azobenzene derivative **1p** was synthesized according to general procedure B3 from 2-aminopyridine and nitrosobenzene, purified by silica gel column chromatography with a hexanes/ethyl acetate (7/3) eluent, and obtained as red solid (1.57 g, 76% yield), m.p. 37 °C. ^{1}H NMR ($CDCl_3$, 400 MHz, 298 K): δ 8.59 (dd, 1H, J = 4.4 Hz, 1.6 Hz), 7.92 (dd, 2H, J = 7.2 Hz, 2.4 Hz), 7.73–7.65 (m, 2H), 7.40–7.35 (m, 3H), 7.23–7.19 (m, 1H) ppm. The obtained spectral data are well-matched to those in the literature.[17]

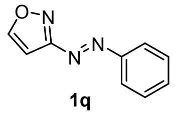

1q

(E)-3-(phenyldiazenyl)isoxazole (1q) The azobenzene derivative **1q** was synthesized according to general procedure B1 from isoxazol-3-amine and nitrosobenzene, purified by silica gel column chromatography with a hexanes/ethyl acetate (8/2) eluent, and obtained as yellow solid (540 mg, 78% yield), m.p. 79 °C. ^{1}H NMR ($CDCl_3$, 400 MHz, 298 K): δ 8.45–8.44 (m, 1H), 7.99–7.96 (m, 2H), 7.53–7.50 (m, 3H), 6.72–6.71 (m, 1H) ppm. ^{13}C{^{1}H} NMR ($CDCl_3$, 101 MHz, 298 K): δ 173.0, 160.2, 152.3, 133.1, 129.3, 123.7, 95.3, 77.5, 77.2, 76.8 ppm. HRMS-ESI$^+$ (m/z): $[M+H]^+$ calcd. For $C_9H_8N_3O^+$, 174.0662; found, 174.0659.

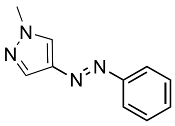

1r

(E)-1-methyl-4-(phenyldiazenyl)-1H-pyrazole (1r) Azobenzene derivative **1r** was synthesized *via* following modification of the literature.[19] An oven-dried 100-mL round bottom flask was charged with nitrosobenzene (490 mg, 4.60 mmol, 1.00 equiv), a solution of 1-methyl-1H-pyrazol-4-amine (440 μL, 5.20 mmol, 1.10 equiv.) in pyridine (3 mL), and 40% aq. NaOH (3 mL). With vigorous stirring, the reaction mixture was heated to 80 °C for 2 h in an oil bath using a hot plate. The reaction mixture was cooled to 23 °C. Water (10 mL) was added. The layers were separated and the aqueous layer was extracted with EtOAc (3 × 25 mL). The combined organic layers were washed with brine (3 × 25 mL) and concentrated under reduced pressure. The crude product was purified by silica gel column chromatography with a hexanes/ethyl acetate (8/2) eluent and obtained as an orange solid (265 mg, 31% yield), m.p. 58 °C. ^{1}H NMR ($CDCl_3$, 400 MHz,

298 K): δ 8.01 (s, 1H), 7.94–7.93 (m, 1H), 7.78 (d, 2H, *J* = 6.8 Hz), 7.46 (t, 2H, *J* = 7.2 Hz), 7.39 (t, 1H, *J* = 7.2 Hz), 3.92 (s, 3H) ppm. The obtained spectral data are well-matched to those in the literature.[18]

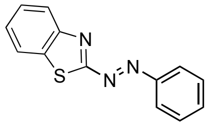

1s

(E)-2-(phenyldiazenyl)benzo[d]thiazole (1s) The azobenzene derivative **1s** was synthesized according to general procedure B1 from benzo[d]thiazol-2-amine and nitrosobenzene, purified by silica gel column chromatography with a hexanes/ethyl acetate (7/3) eluent, and obtained as orange crystalline solid (680 mg, 71% yield), m.p. 149 °C. ^{1}H NMR ($CDCl_3$, 400 MHz, 298 K): δ 8.19 (d, 1H, *J* = 7.2 Hz), 7.08 (dd, 2H, *J* = 2.0 Hz, 7.2 Hz), 7.90 (d, 1H, *J* = 7.6 Hz), 7.59–7.52 (m, 4H), 7.47 (dt, 1H, *J* = 1.6 Hz, 7.6 Hz) ppm. The obtained spectral data are well-matched to those in the literature.[19]

C. Supporting Data

a)

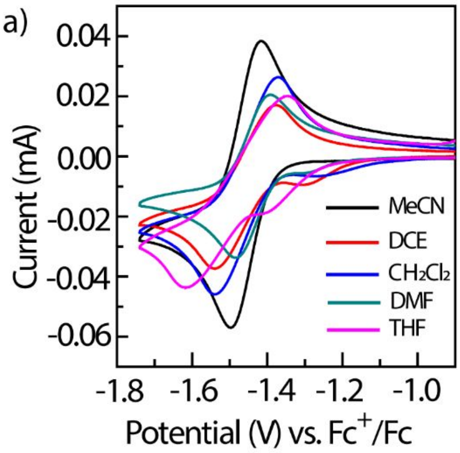

b)

Solvent	Peak to peak separation (mV)	Peak current ratio
MeCN	80	0.99
DMF	94	0.99
CH_2Cl_2	170	0.94
DCE	163	0.72
THF	271	0.86

Figure S1. Solvent optimization for electrochemical characterization of 1a. a) CVs of a 0.005 M solution of **1a** in MeCN, DCE, CH_2Cl_2 DMF, and THF using 0.1 M $TBAPF_6$ as supporting electrolyte (potentials *vs.* Fc^+/Fc), CV plotting follows IUPAC convention, starting from 0 V and follows reductive direction with a scan rate of 0.1 V/s, b) Comparison of electrochemical parameters measured in different organic solvents.

a)

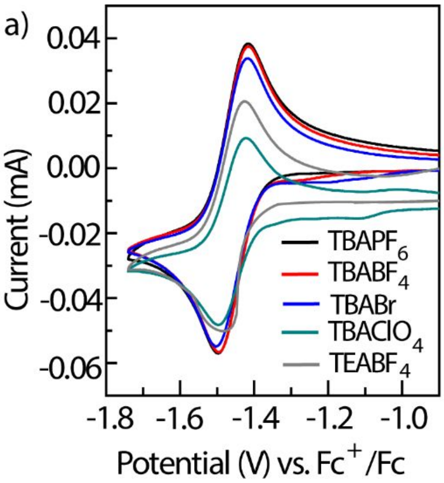

b)

Electrolyte	Peak to peak separation (mV)	Peak current ratio
$TBAPF_6$	80	0.99
$TBABF_4$	81	0.96
TBABr	85	0.96
$TBAClO_4$	74	0.96
$TEABF_4$	73	0.93

Figure S2. Optimization of supporting electrolyte for electrochemical characterization of 1a. a) CVs of a 0.005 M solution of **1a** in MeCN using 0.1 M $TBAPF_6$, $TBABF_4$, TBABr, $TBAClO_4$, $TEABF_4$ as supporting electrolyte (potentials *vs.* Fc^+/Fc), CV plotting follows IUPAC convention, starting from 0 V and follows reductive direction with a scan rate of 0.1 V/s, b) Comparison of electrochemical parameters between different supporting electrolytes.

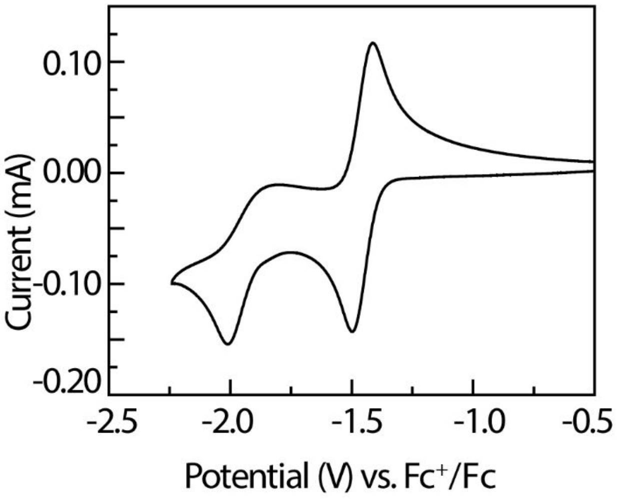

Figure S3. a) CV of a 0.005 M solution of **1a** in MeCN using 0.1 M $TBAPF_6$ as supporting electrolyte (potentials *vs.* Fc^+/Fc), CV plotting follows IUPAC convention, starting from 0 V and follows reductive direction with a scan rate of 0.1 V/s.

Cycling capacity of 1a in cyclic voltammetry

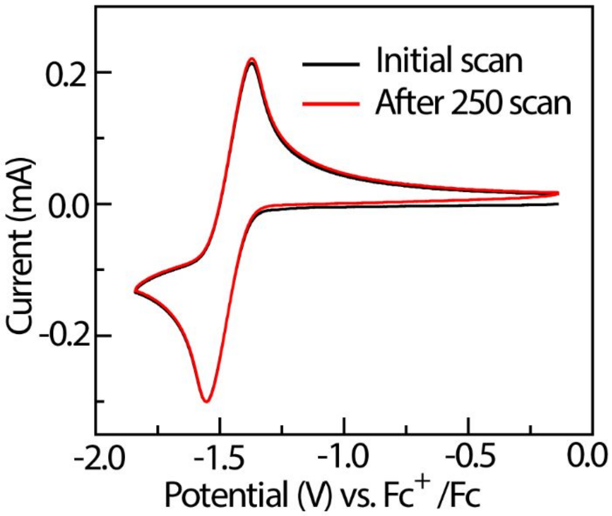

Figure S4. CVs of a 0.005 M solution of **1a** in MeCN using 0.1 M $TBAPF_6$ as supporting electrolyte before and after 250 scans (potentials *vs.* Fc^+/Fc), CV plotting follows IUPAC convention, starting from 0 V and follows reductive direction with a scan rate of 0.1 V/s.

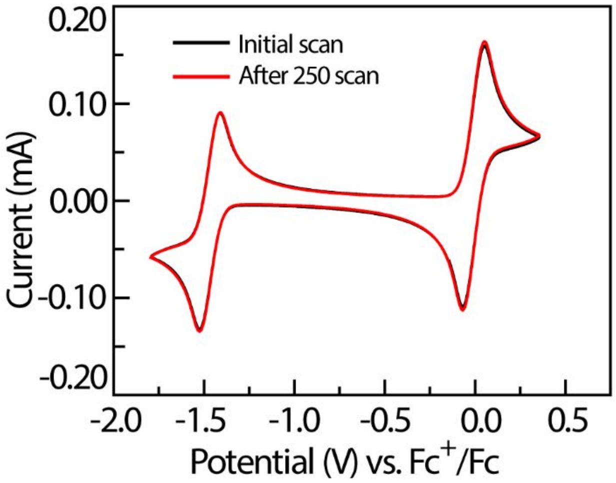

Figure S5. CVs of a 0.005 M solution of **1a** in MeCN using 0.1 M $TBAPF_6$ as supporting electrolyte before and after 250 scans in presence of ferrocene (potentials *vs.* Fc^+/Fc), CV plotting follows IUPAC convention, starting from 0 V and follows reductive direction with a scan rate of 0.1 V/s.

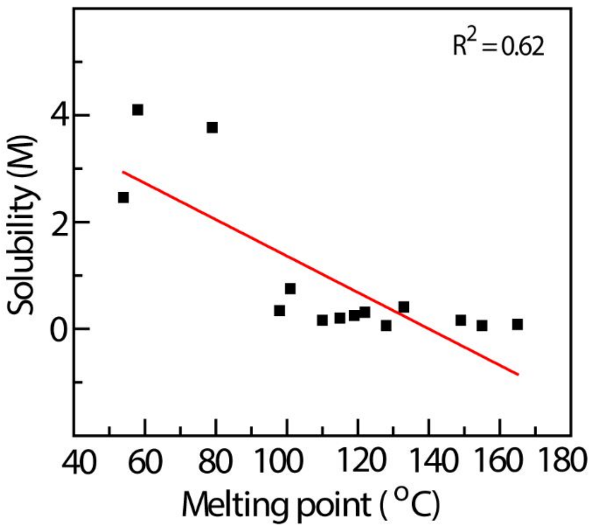

Figure S6. Correlation between solubility and melting point for the azobenzene derivatives. Azobenzene derivatives that are liquidous at ambient temperature (*i.e.*, MP < 23 °C) were found to be completely miscible in MeCN.

Cycling capacity of 1i by cyclic voltammetry

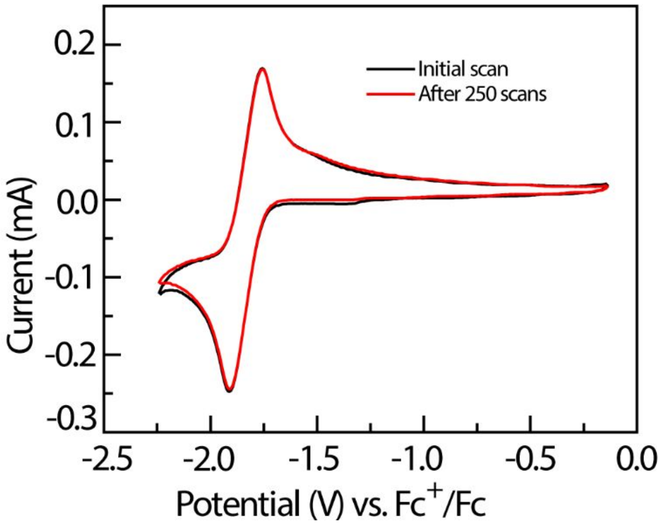

Figure S7. CVs of a 0.005 M solution of **1i** in MeCN using 0.1 M $TBAPF_6$ as supporting electrolyte before and after 250 scans in CV (potentials *vs.* Fc^+/Fc), CV plotting follows IUPAC convention, starting from 0 V and follows reductive direction with a scan rate of 0.1 V/s.

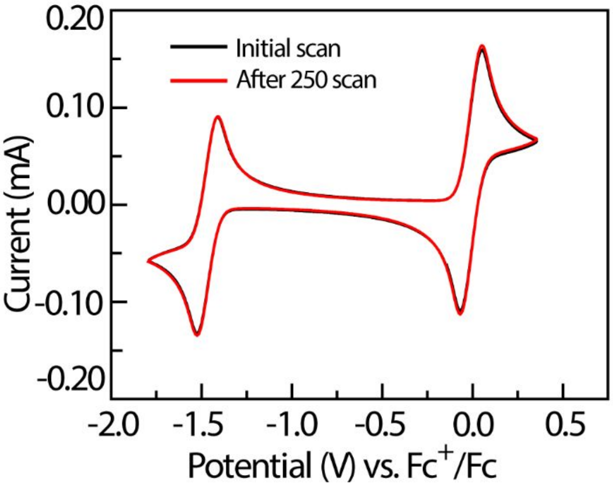

Figure S8. CVs of a 0.005 M solution of **1i** in MeCN using 0.1 M $TBAPF_6$ as supporting electrolyte before and after 250 scans in presence of ferrocene (Fc^+/Fc was used as reference), CV plotting follows IUPAC convention, starting from 0 V and follows reductive direction with a scan rate of 0.1 V/s.

Estimation of Diffusion Coefficient

The Randles-Ševčík equation was used to determine the diffusion coefficient (D) (eqn. 1). A plot of cathodic and anodic peak current *vs*. square root of scan rate is linear (Figure S7), which indicates electrochemical reversibility and a diffusion limited process. This equation was used to obtain the diffusion coefficient (D).

$$\text{Peak current, } i_p = 0 \cdot 4463nFAC\sqrt{\frac{nFvD}{RT}} \text{ ……………………. (eqn. 1)}$$

For equation 1, i_p is the peak current (A), n is the number of electrons transferred, F is Faraday constant (96485 C/mol e^-), A is the electrode area (0.071 cm^2), C is the bulk concentration of redox active species in mol/cm^3, R is the universal gas constant (8.3145 J/mol•K), T is the temperature in K, υ is the scan rate in V/s, D is the diffusion coefficient in (cm^2/s).

Table S1. Comparison of electrochemical parameters of different azobenzene derivatives.

	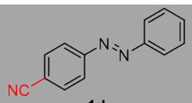 **1d**	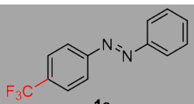 **1e**	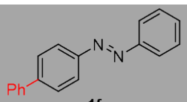 **1f**	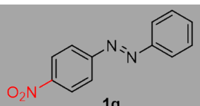 **1g**	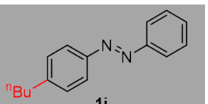 **1i**
$E_{1/2}$ (V)	−1.47	−1.57	−1.72	−1.25, −1.59	−1.84
State at 298 K	solid	solid	solid	solid	liquid
Diffusion coefficient (cm^2/s):	1.23 × 10^{-5}	1.13 × 10^{-5}	1.12 × 10^{-5}	1.08 × 10^{-5}	1.25 × 10^{-5}
Theoretical capacity (AhL^{-1})	24.4	21.9	24.1	26.8	53.6

Characterization of monoreduced species by UV-vis spectroscopy and degradation pathway

*Monoreduction of **1a**.* A solution of **1a** (5 mM) containing 0.1 M $TBAPF_6$ (purchased from Oakwood Chemicals) was prepared in dry MeCN and transferred to an electrochemical cell under N_2 atmosphere. Bulk electrolysis experiment was carried out at 23 °C using CH Instruments Electrochemical Analyzer (Model CHI620A) in a three-electrode cell using planner Pt working electrode, planner Pt counter electrode, Ag-reference electrode obtained from IKA, the distance between working and counter electrode is 3.5 mm in 5-mL Electrasyn glass vial. Reference electrode was prepared using 0.1 M solution of $TBAPF_6$ in acetonitrile with 1.0 mM $AgNO_3$. A constant potential of –1.54 V *vs*. Fc^+/Fc was applied until the color of the solution turned from orange to red during 1.5 hours; a total applied charge of 1 F/mole generated the monoreduced product, **1a′**. UV-vis spectra were obtained after transferring the solution to a screw cap quartz cell under N_2 atmosphere.

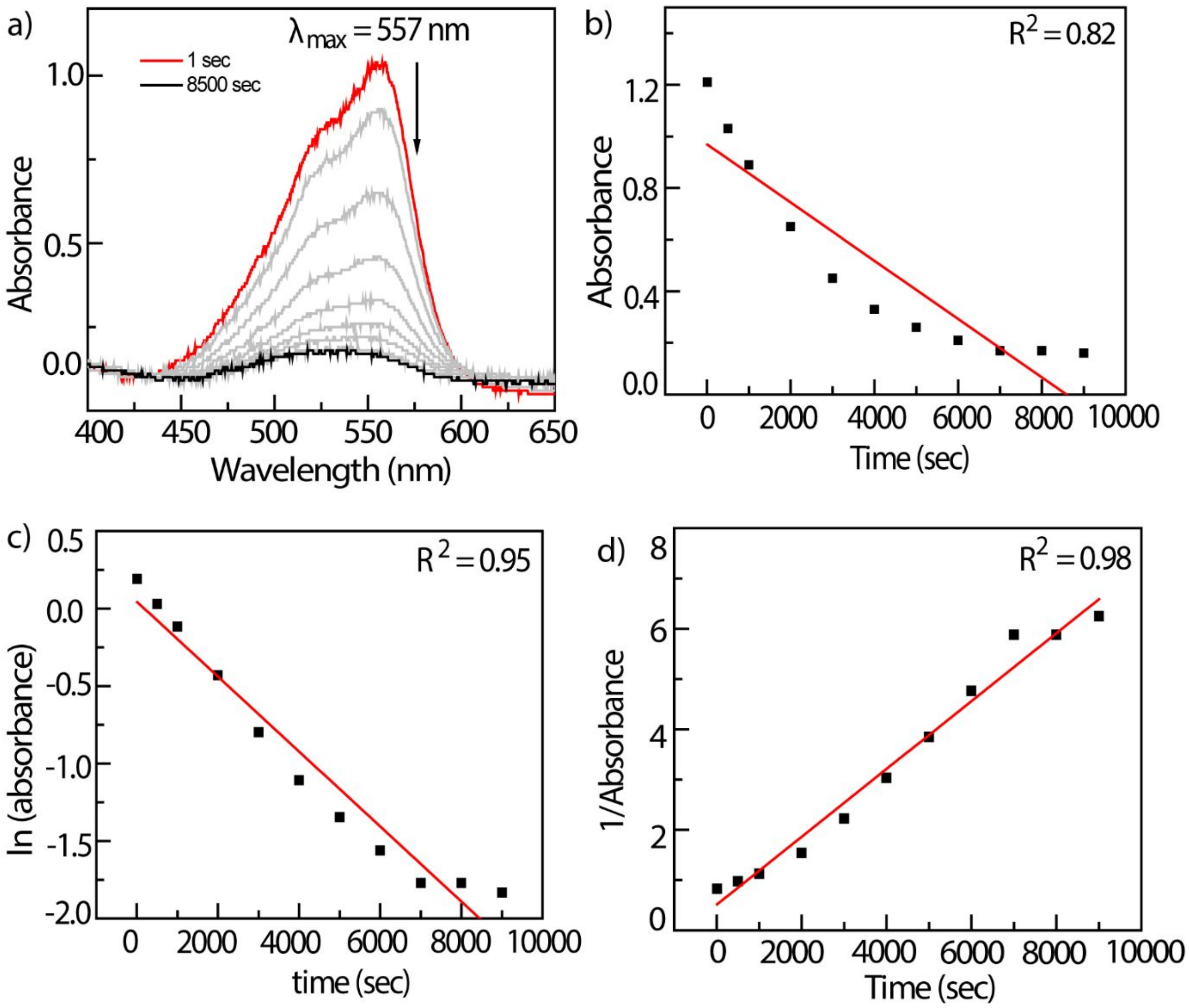

Figure S9. a) UV-vis spectra depicting the degradation of monoreduced species **1a′** after bulk electrolysis of **1a** obtained over 8500 sec. b) Plot of Absorbance (■) at 557 nm and linear fit (—) *vs*. time. c) Plot of ln(Absorbance) (■) and linear fit (—) *vs.* time. d) Plot of (1/Absorbance) (■) and linear fit (—) *vs.* time.

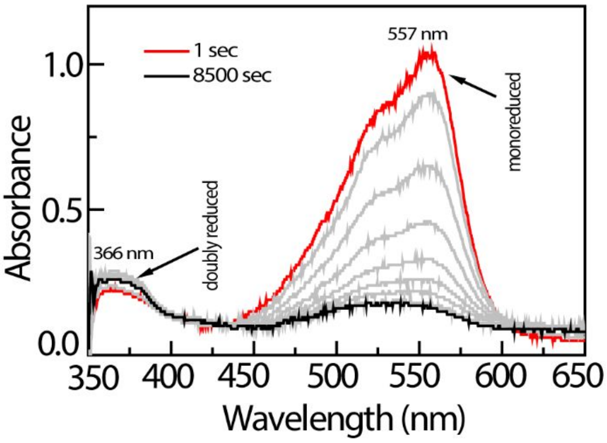

Figure S10. UV-vis spectra obtained during the electrosynthesis of the doubly reduced dianion derived from **1a**. Electrolysis carried out at a constant potential –1.54 V *vs.* Fc^{+}/Fc in MeCN.

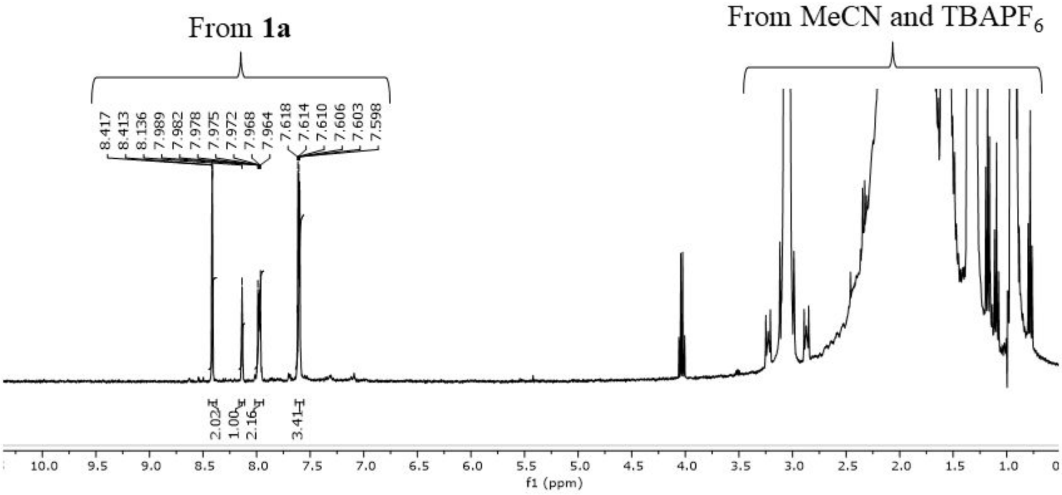

Figure S11. ^{1}H NMR spectrum obtained after bulk electrolysis of **1a** at –1.54 V *vs.* Fc^{+}/Fc in MeCN. NMR taken in CD_3CN at 298 K and 400 MHz.

Plausible Mechanism for Degradation of Monoreduced Azobenzene Derivative

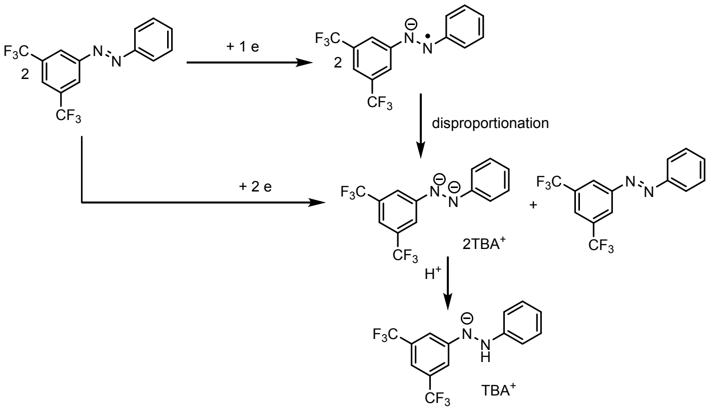

Scheme S1. Plausible mechanism for the degradation of azobenzene radical anion through disproportionation.[20-21]

Evaluation of the Water Sensitivity of the Reductive Chemistry of 1g

a)

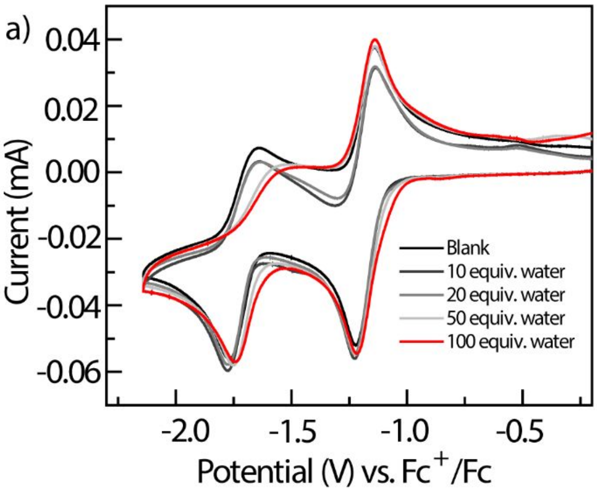

b)

H_2O equiv	i_{pa1}/i_{pc1}	i_{pa2}/i_{pc2}
Blank	0.84	0.69
10	0.82	0.66
20	0.77	0.59
50	0.73	-
100	0.70	-

Figure S12. a) CVs of a 0.005 M solution of **1g** in MeCN with 0.1 M $TBAPF_6$ as supporting electrolyte in the absence of water and then upon gradual increase of water (potentials *vs.* Fc^+/Fc), CV plotting follows IUPAC convention, starting from 0 V and follows reductive direction with a scan rate of 0.1 V/s, b) Dependence of anodic to cathodic peak current ratio on water.

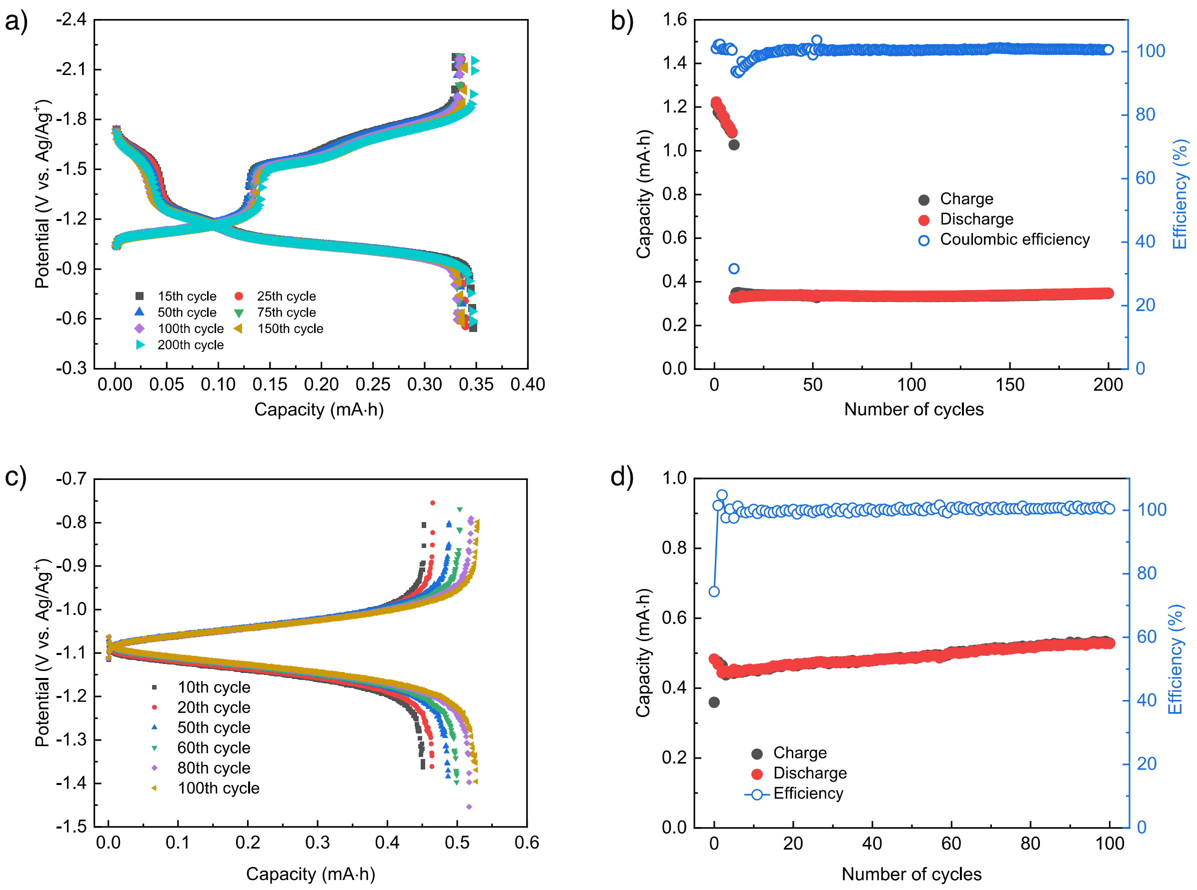

Figure S13. Galvastonic charge–discharge cycles of 1h. a) Charge–discharge curves of **1h** (5 mM) measured at 5 mA constant current with carbon electrodes in a symmetrical H-cell. Both reduction events sampled in this experiment. b) Cycled capacity and measured coulombic efficiency for **1h** in a H-cell. Both reduction events sampled in this experiment. c) Charge–discharge curves of **1h** (5 mM) measured at 5 mA constant current with carbon electrodes in a symmetrical H-cell. Only the first reduction event was sampled in this experiment. d) Cycled capacity and measured coulombic efficiency for **1h** in a H-cell. Only the first reduction event was sampled in this experiment.

D. Electrochemical and Spectroscopic Characterization

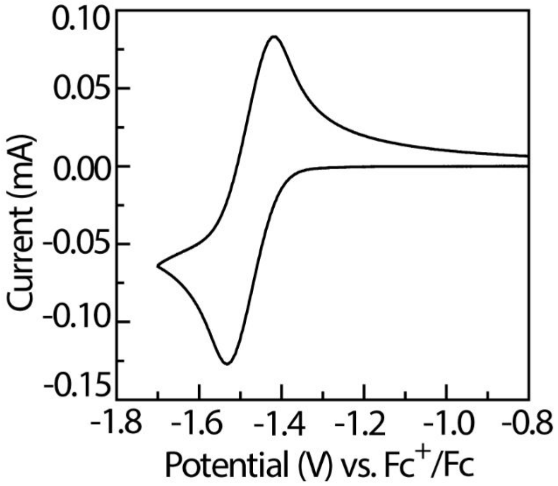

Figure S14. CV of a 0.005 M solution of **1a** in acetonitrile with 0.1 M $TBAPF_6$ supporting electrolyte. Data measured with a glassy carbon working electrode, CV plotting follows IUPAC convention, starting from 0 V and follows reductive direction with a scan rate of 0.1 V/s.

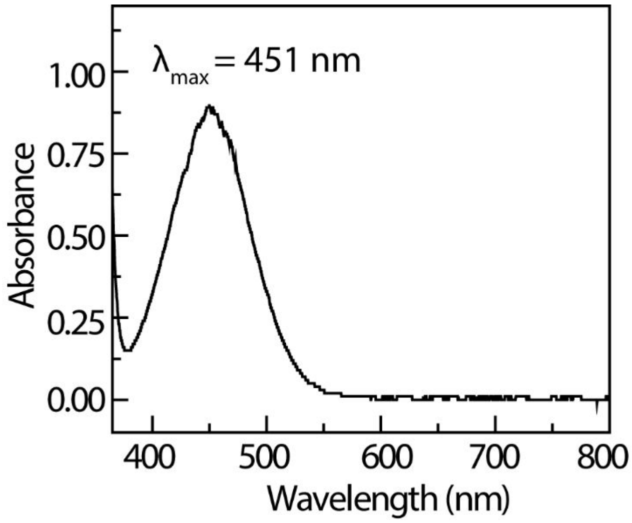

Figure S15. UV-vis spectrum of 2 mM solution of **1a** in MeCN.

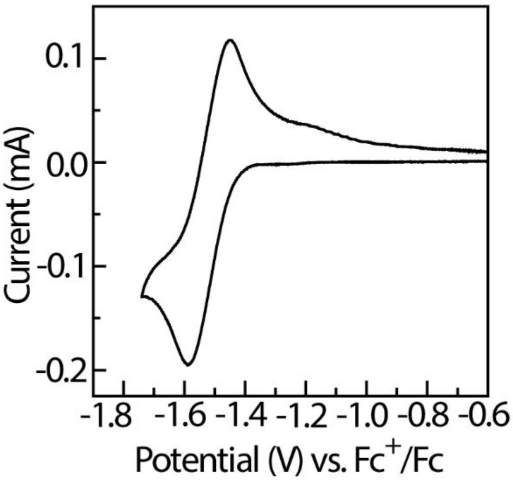

Figure S16. CV of a 0.005 M solution of **1b** in acetonitrile with 0.1 M $TBAPF_6$ supporting electrolyte. Data measured with a glassy carbon working electrode, CV plotting follows IUPAC convention, starting from 0 V and follows reductive direction with a scan rate of 0.1 V/s.

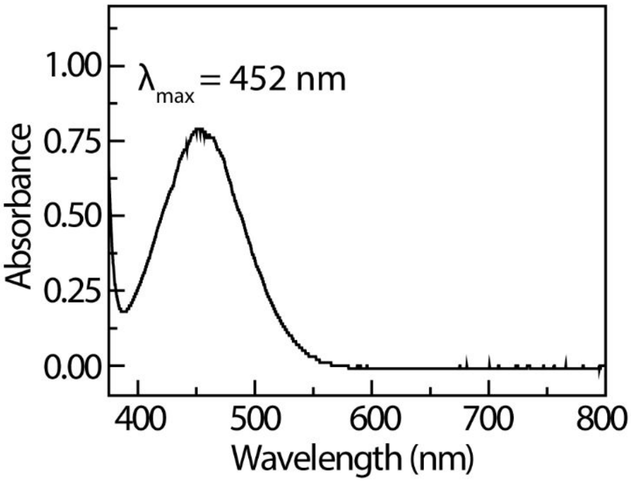

Figure S17. UV-vis spectrum of 2 mM solution of **1b** in MeCN.

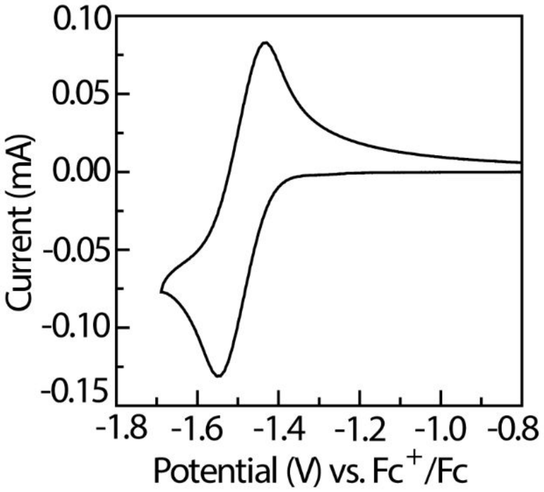

Figure S18. CV of a 0.005 M solution of **1c** in acetonitrile with 0.1 M $TBAPF_6$ supporting electrolyte. Data measured with a glassy carbon working electrode, CV plotting follows IUPAC convention, starting from 0 V and follows reductive direction with a scan rate of 0.1 V/s.

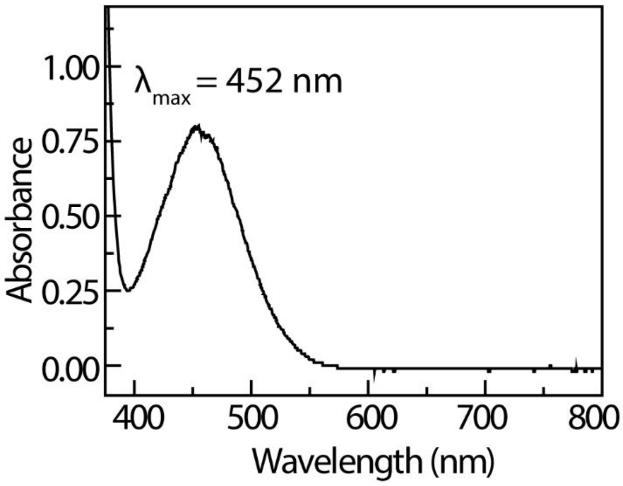

Figure S19. UV-vis spectrum of 2 mM solution of **1c** in MeCN.

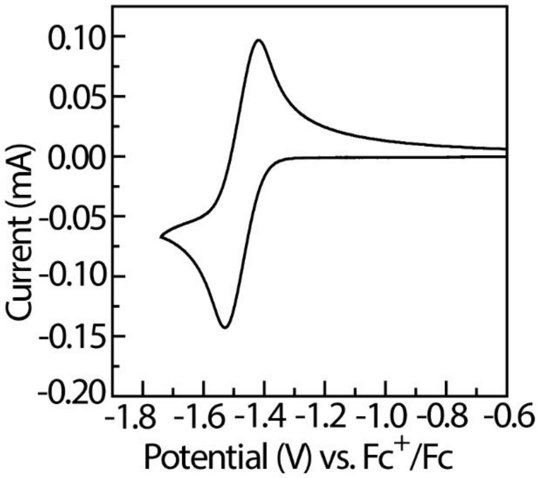

Figure S20. CV of a 0.005 M solution of **1d** in acetonitrile with 0.1 M $TBAPF_6$ supporting electrolyte. Data measured with a glassy carbon working electrode, CV plotting follows IUPAC convention, starting from 0 V and follows reductive direction with a scan rate of 0.1 V/s.

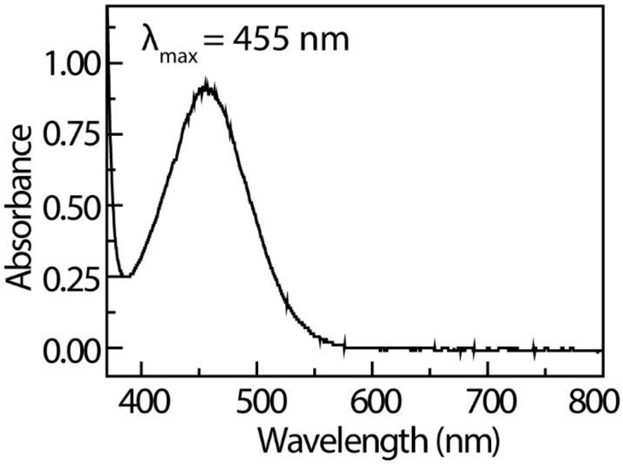

Figure S21. UV-vis spectrum of 2 mM solution of **1d** in MeCN.

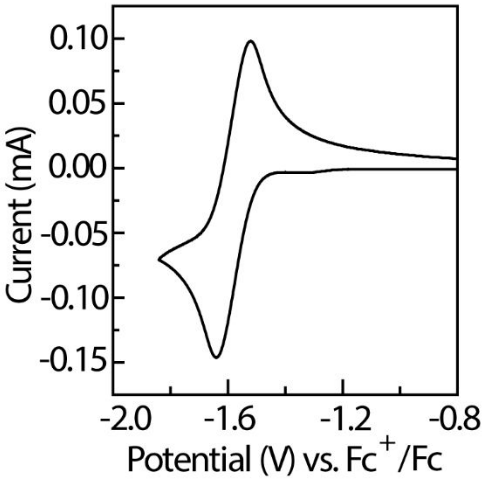

Figure S22. CV of a 0.005 M solution of **1e** in acetonitrile with 0.1 M $TBAPF_6$ supporting electrolyte. Data measured with a glassy carbon working electrode, CV plotting follows IUPAC convention, starting from 0 V and follows reductive direction with a scan rate of 0.1 V/s.

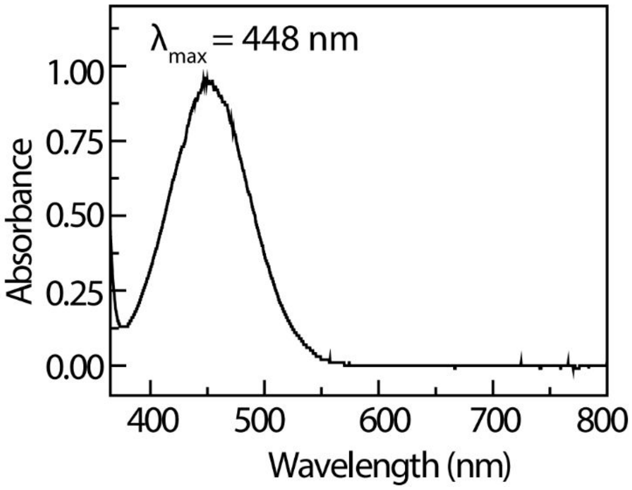

Figure S23. UV-vis spectrum of 2 mM solution of **1e** in MeCN.

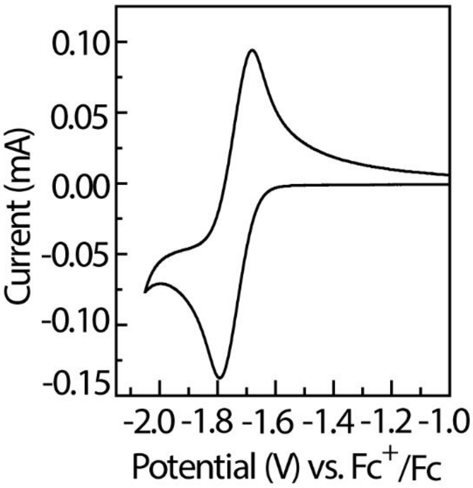

Figure S24. CV of a 0.005 M solution of **1f** in acetonitrile with 0.1 M $TBAPF_6$ supporting electrolyte. Data measured with a glassy carbon working electrode, CV plotting follows IUPAC convention, starting from 0 V and follows reductive direction with a scan rate of 0.1 V/s.

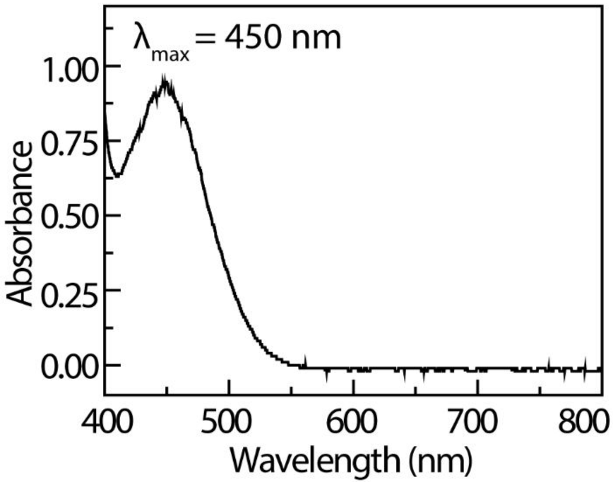

Figure S25. UV-vis spectrum of 2 mM solution of **1f** in MeCN.

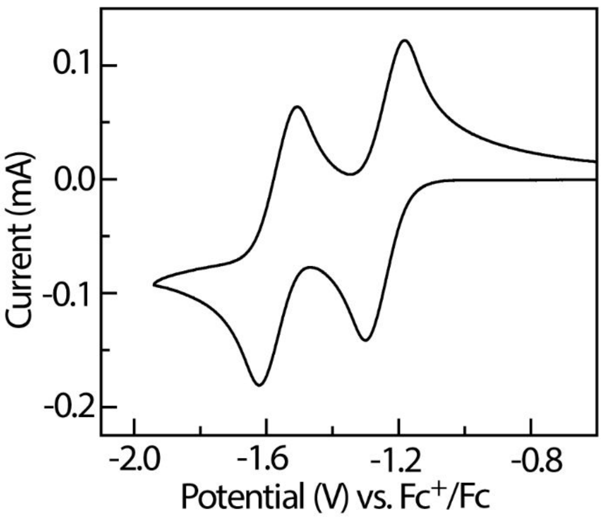

Figure S26. CV of a 0.005 M solution of **1g** in acetonitrile with 0.1 M $TBAPF_6$ supporting electrolyte. Data measured with a glassy carbon working electrode, CV plotting follows IUPAC convention, starting from 0 V and follows reductive direction with a scan rate of 0.1 V/s.

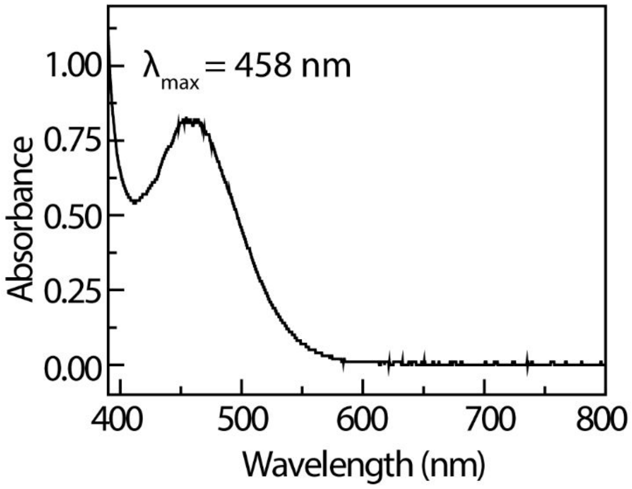

Figure S27. UV-vis spectrum of 2 mM solution of **1g** in MeCN.

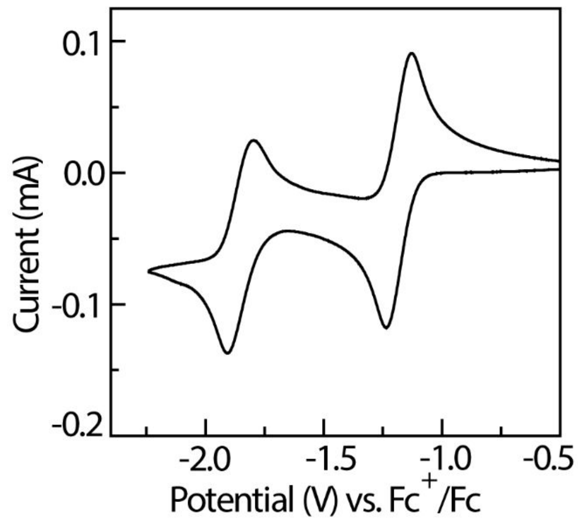

Figure S28. CV of a 0.005 M solution of **1h** in acetonitrile with 0.1 M $TBAPF_6$ supporting electrolyte. Data measured with a glassy carbon working electrode, CV plotting follows IUPAC convention, starting from 0 V and follows reductive direction with a scan rate of 0.1 V/s. We believe that the difference in peak heights of the first and second peak is due to the diffusion of the radical anion during the CV time scale.

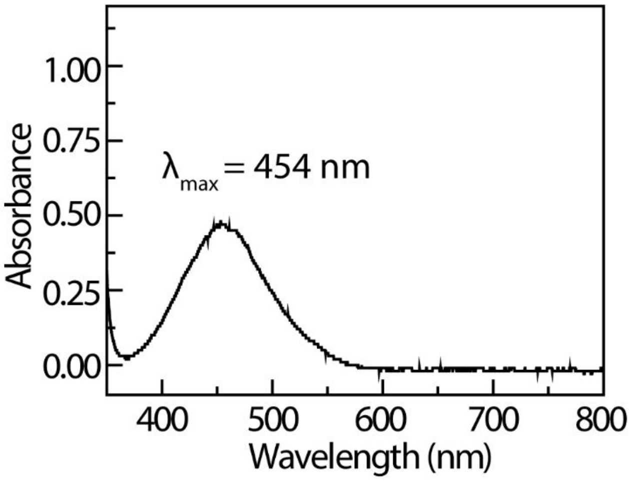

Figure S29. UV-vis spectrum of 2 mM solution of **1h** in MeCN.

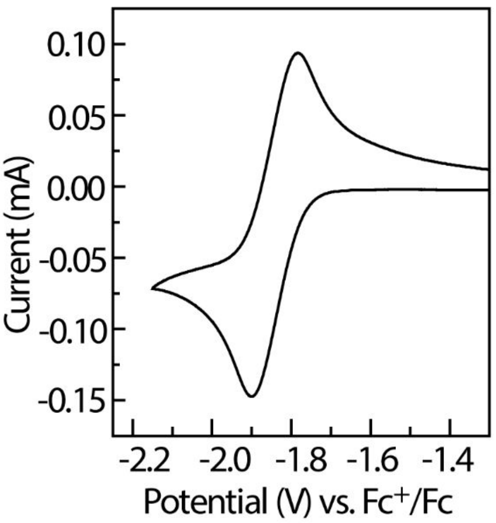

Figure S30. CV of a 0.005 M solution of **1i** in acetonitrile with 0.1 M $TBAPF_6$ supporting electrolyte. Data measured with a glassy carbon working electrode, CV plotting follows IUPAC convention, starting from 0 V and follows reductive direction with a scan rate of 0.1 V/s.

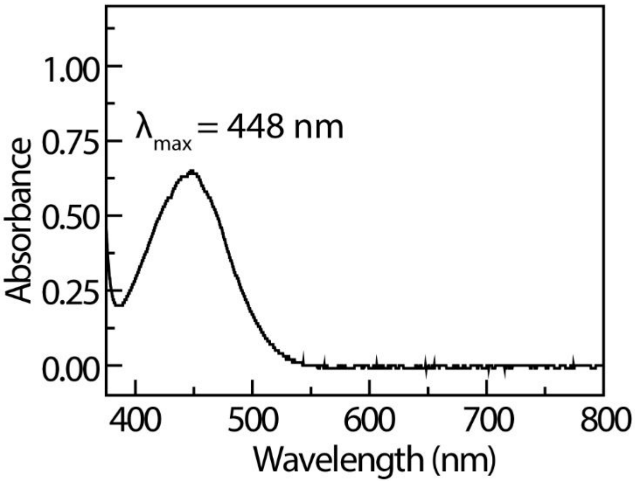

Figure S31. UV-vis spectrum of 2 mM solution of **1i** in MeCN.

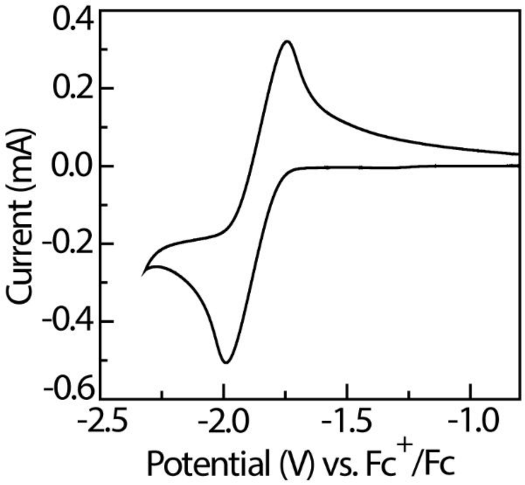

Figure S32. CV of a 0.005 M solution of **1j** in acetonitrile with 0.1 M $TBAPF_6$ supporting electrolyte. Data measured with a glassy carbon working electrode, CV plotting follows IUPAC convention, starting from 0 V and follows reductive direction with a scan rate of 0.1 V/s.

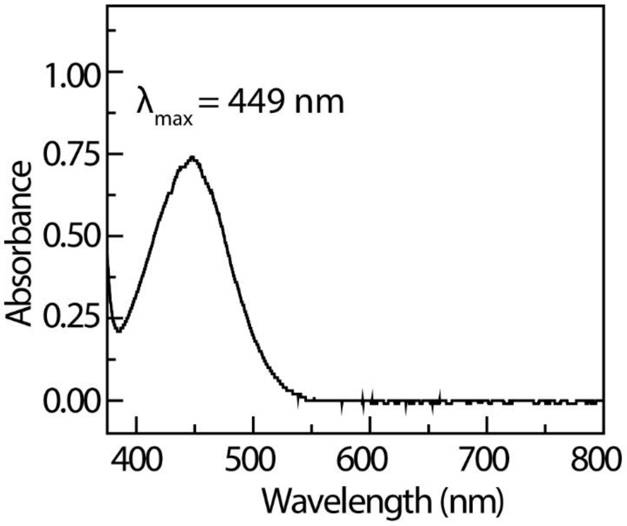

Figure S33. UV-vis spectrum of 2 mM solution of **1j** in MeCN.

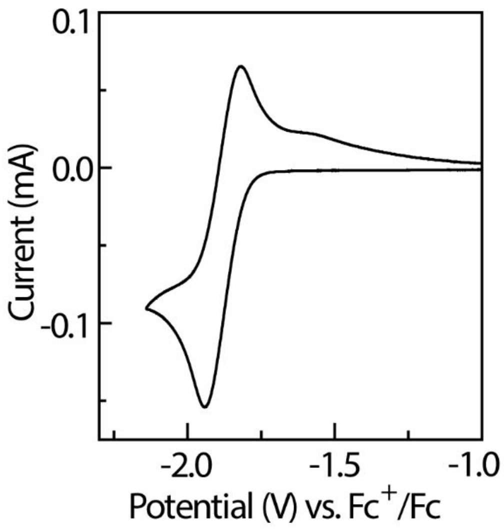

Figure S34. CV of a 0.005 M solution of **1k** in acetonitrile with 0.1 M $TBAPF_6$ supporting electrolyte. Data measured with a glassy carbon working electrode, CV plotting follows IUPAC convention, starting from 0 V and follows reductive direction with a scan rate of 0.1 V/s.

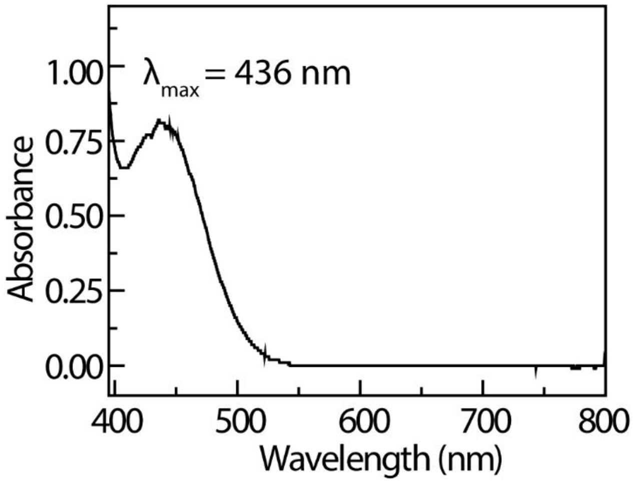

Figure S35. UV-vis spectrum of 2 mM solution of **1k** in MeCN.

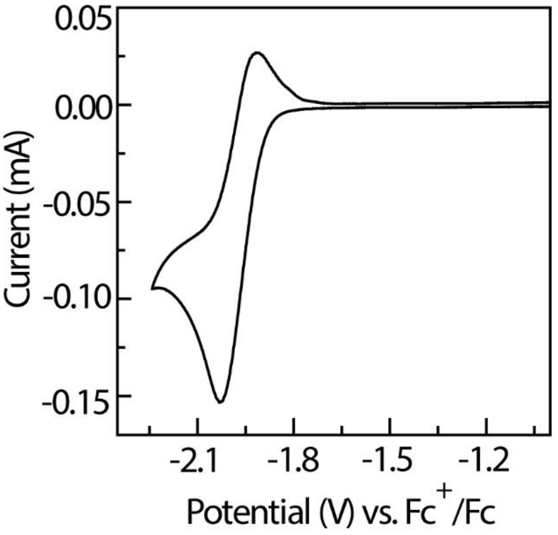

Figure S36. CV of a 0.005 M solution of **1l** in acetonitrile with 0.1 M $TBAPF_6$ supporting electrolyte. Data measured with a glassy carbon working electrode, CV plotting follows IUPAC convention, starting from 0 V and follows reductive direction with a scan rate of 0.1 V/s.

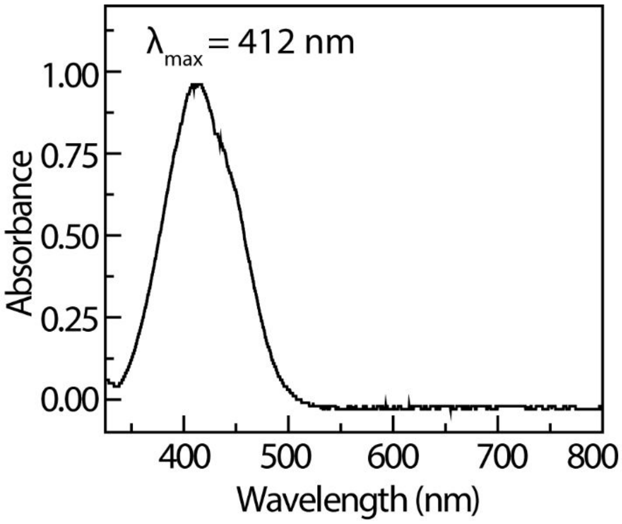

Figure S37. UV-vis spectrum of 2 mM solution of **1l** in MeCN.

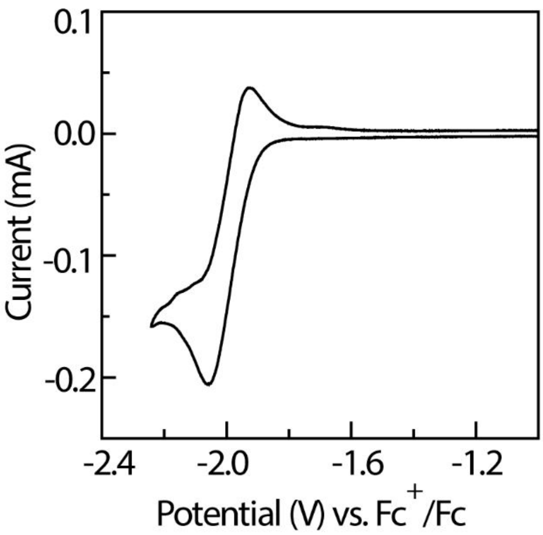

Figure S38. CV of a 0.005 M solution of **1m** in acetonitrile with 0.1 M $TBAPF_6$ supporting electrolyte. Data measured with a glassy carbon working electrode, CV plotting follows IUPAC convention, starting from 0 V and follows reductive direction with a scan rate of 0.1 V/s.

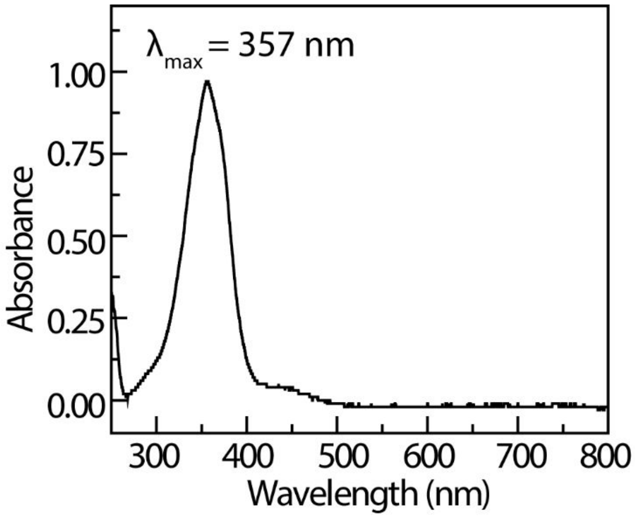

Figure S39. UV-vis spectrum of 2 mM solution of **1m** in MeCN.

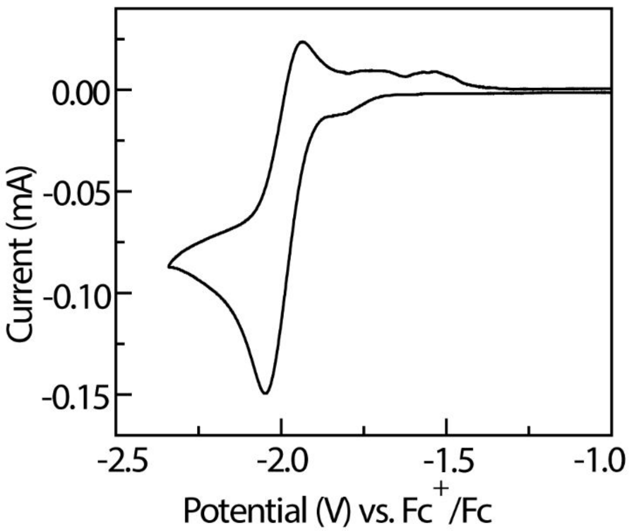

Figure S40. CV of a 0.005 M solution of **1n** in acetonitrile with 0.1 M $TBAPF_6$ supporting electrolyte. Data measured with a glassy carbon working electrode, CV plotting follows IUPAC convention, starting from 0 V and follows reductive direction with a scan rate of 0.1 V/s.

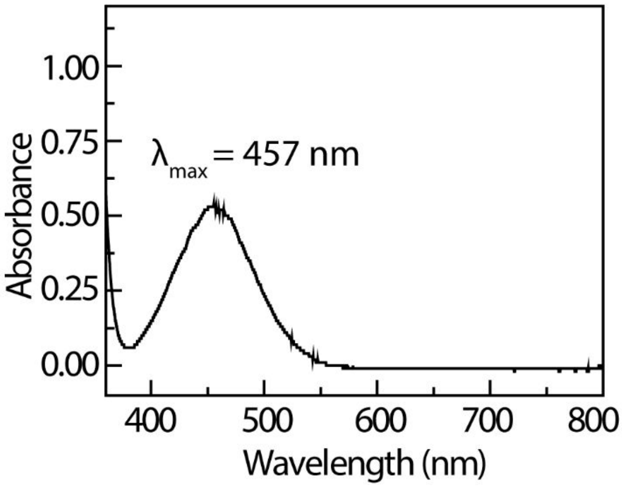

Figure S41. UV-vis spectrum of 2 mM solution of **1n** in MeCN.

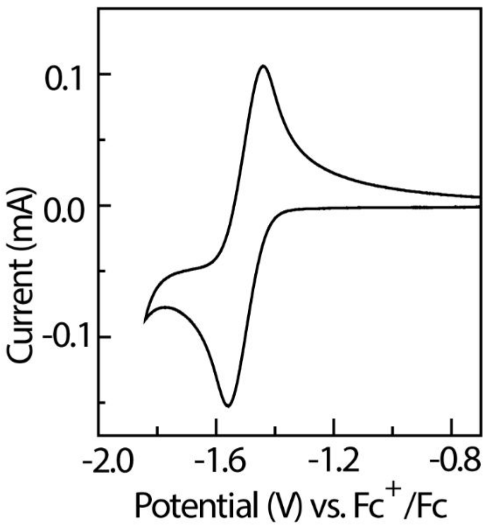

Figure S42. CV of a 0.005 M solution of **1o** in acetonitrile with 0.1 M $TBAPF_6$ supporting electrolyte. Data measured with a glassy carbon working electrode, CV plotting follows IUPAC convention, starting from 0 V and follows reductive direction with a scan rate of 0.1 V/s.

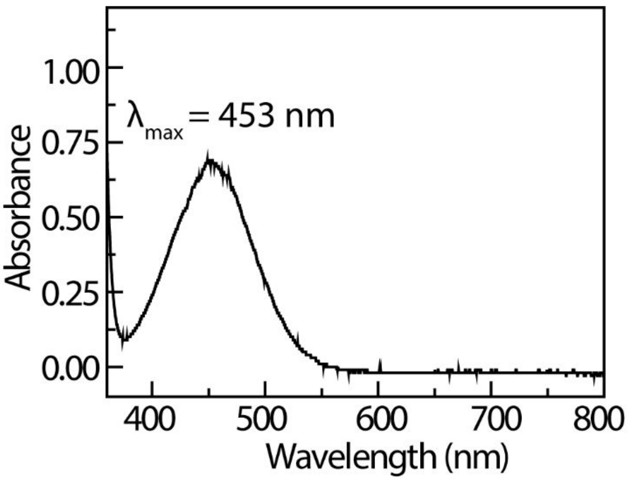

Figure S43. UV-vis spectrum of 2 mM solution of **1o** in MeCN.

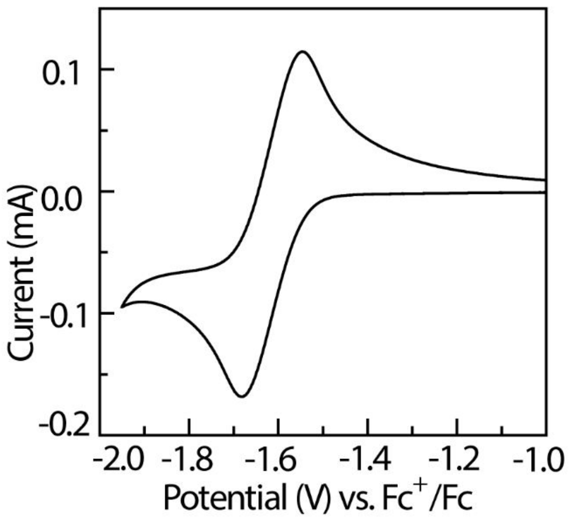

Figure S44. CV of a 0.005 M solution of **1p** in acetonitrile with 0.1 M $TBAPF_6$ supporting electrolyte. Data measured with a glassy carbon working electrode, CV plotting follows IUPAC convention, starting from 0 V and follows reductive direction with a scan rate of 0.1 V/s.

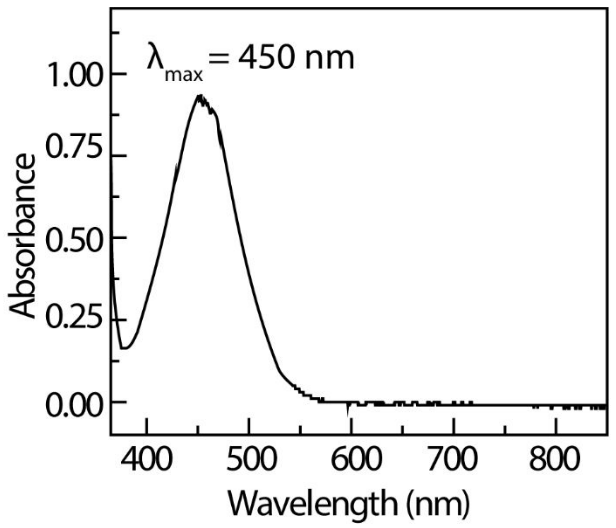

Figure S45. UV-vis spectrum of 2 mM solution of **1p** in MeCN.

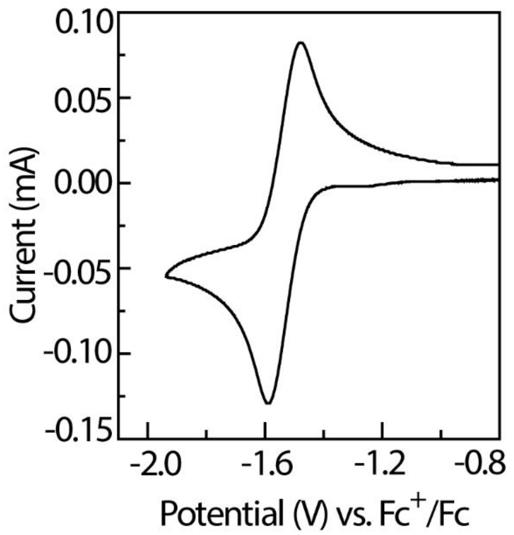

Figure S46. CV of a 0.005 M solution of **1q** in acetonitrile with 0.1 M $TBAPF_6$ supporting electrolyte. Data measured with a glassy carbon working electrode, CV plotting follows IUPAC convention, starting from 0 V and follows reductive direction with a scan rate of 0.1 V/s.

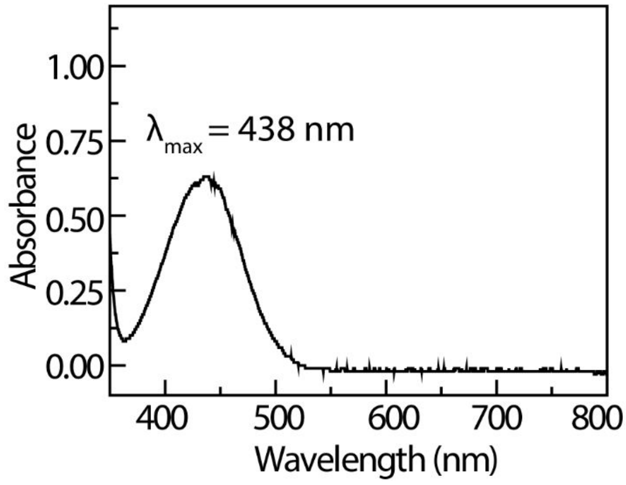

Figure S47. UV-vis spectrum of 2 mM solution of **1q** in MeCN.

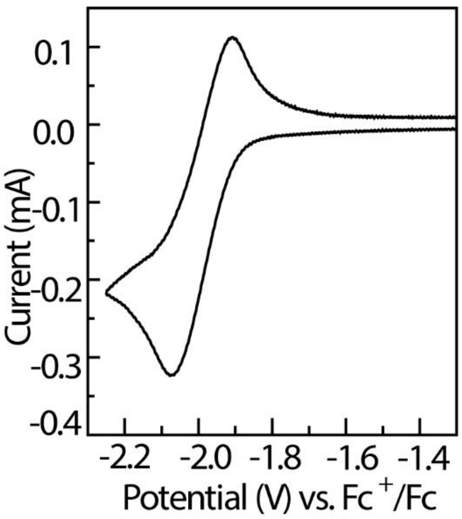

Figure S48. CV of a 0.005 M solution of **1r** in acetonitrile with 0.1 M $TBAPF_6$ supporting electrolyte. Data measured with a glassy carbon working electrode, CV plotting follows IUPAC convention, starting from 0 V and follows reductive direction with a scan rate of 0.1 V/s.

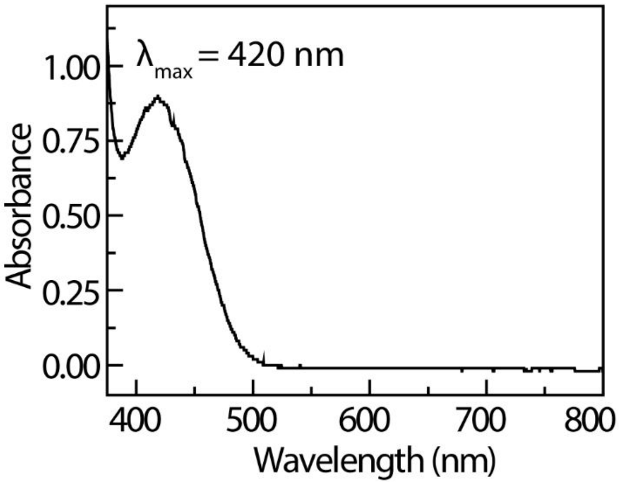

Figure S49. UV-vis spectrum of 2 mM solution of **1r** in MeCN.

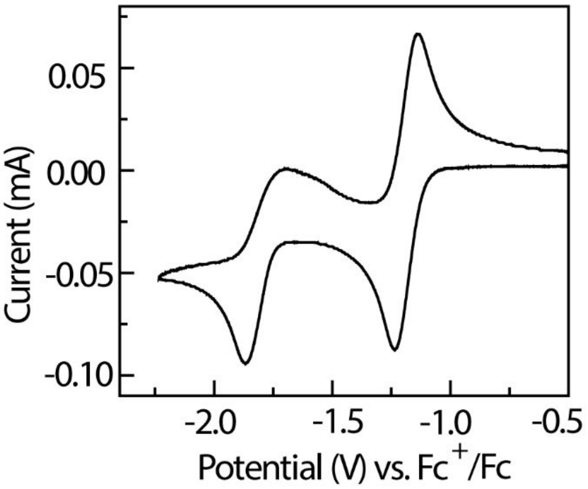

Figure S50. CV of a 0.005 M solution of **1s** in acetonitrile with 0.1 M $TBAPF_6$ supporting electrolyte. Data measured with a glassy carbon working electrode, CV plotting follows IUPAC convention, starting from 0 V and follows reductive direction with a scan rate of 0.1 V/s.

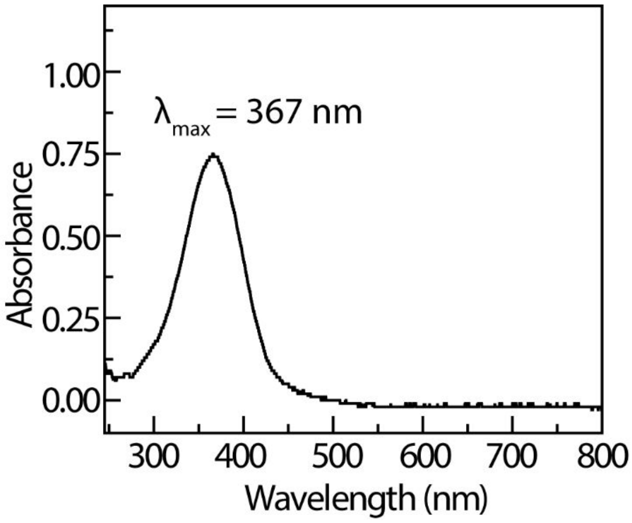

Figure S51. UV-vis spectrum of 2 mM solution of **1s** in MeCN.

E. NMR Spectra of New Compounds

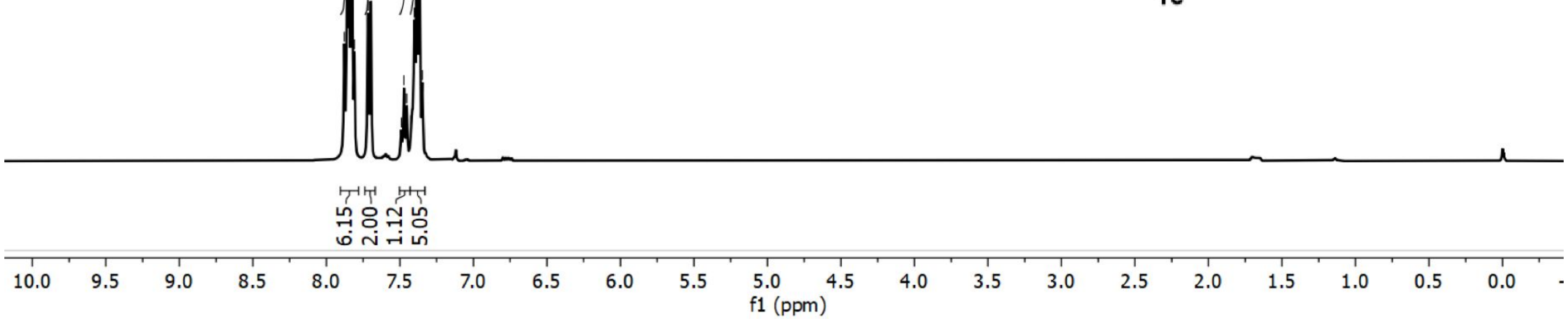

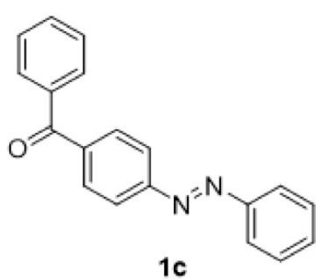

1c

Figure S52. ^{1}H NMR spectrum of (*E*)-phenyl(4-(phenyldiazenyl)phenyl)methanoneone (**1c**) measured in $CDCl_3$, 400 MHz, 298 K.

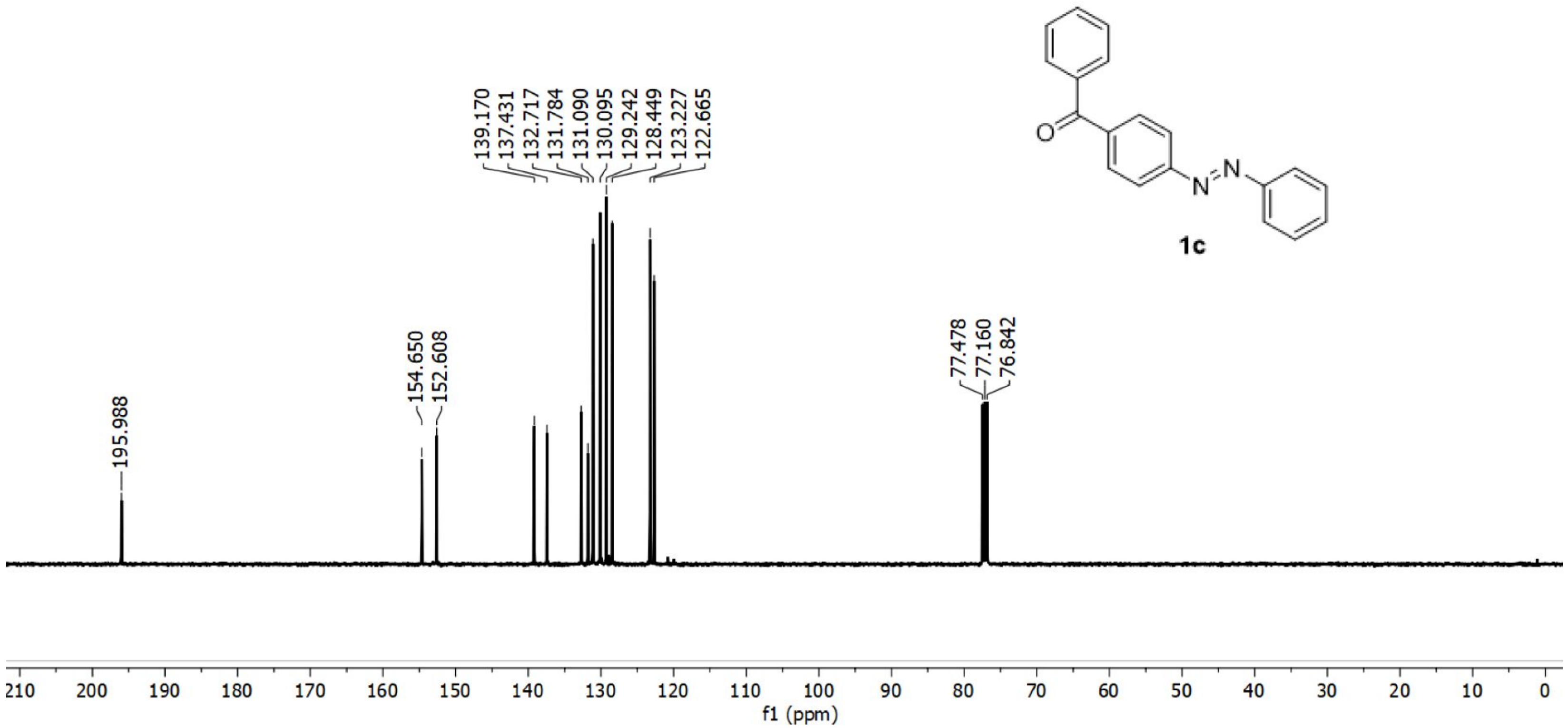

Figure S53. $^{13}C\{^{1}H\}$ NMR spectrum of (*E*)-phenyl(4-(phenyldiazenyl)phenyl)methanoneone (**1c**) measured in $CDCl_3$, 101 MHz, 298 K.

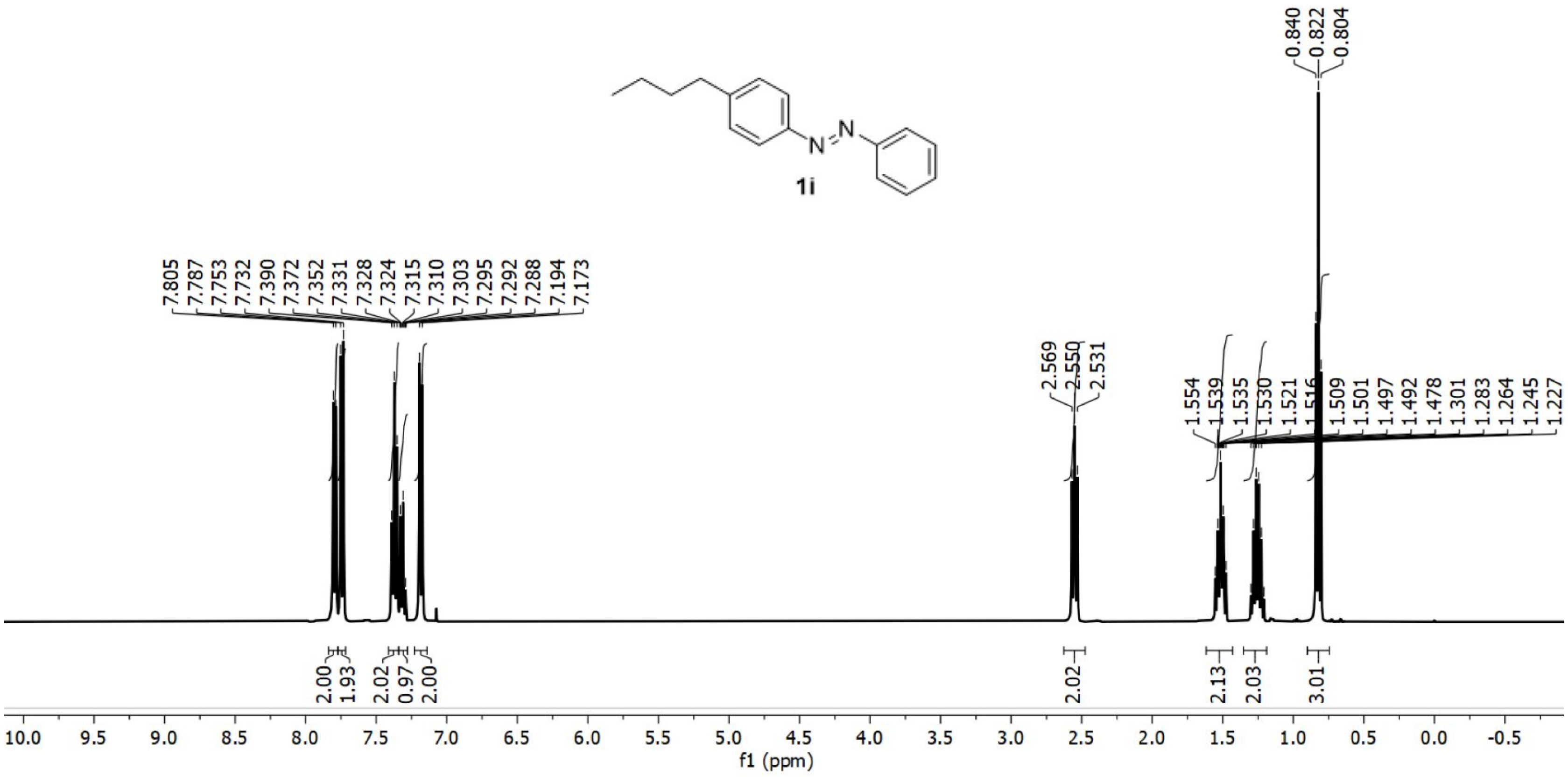

Figure S54. ^{1}H NMR spectrum of (*E*)-1-(4-butylphenyl)-2-phenyldiazene (**1i**) measured in $CDCl_3$, 400 MHz, 298 K.

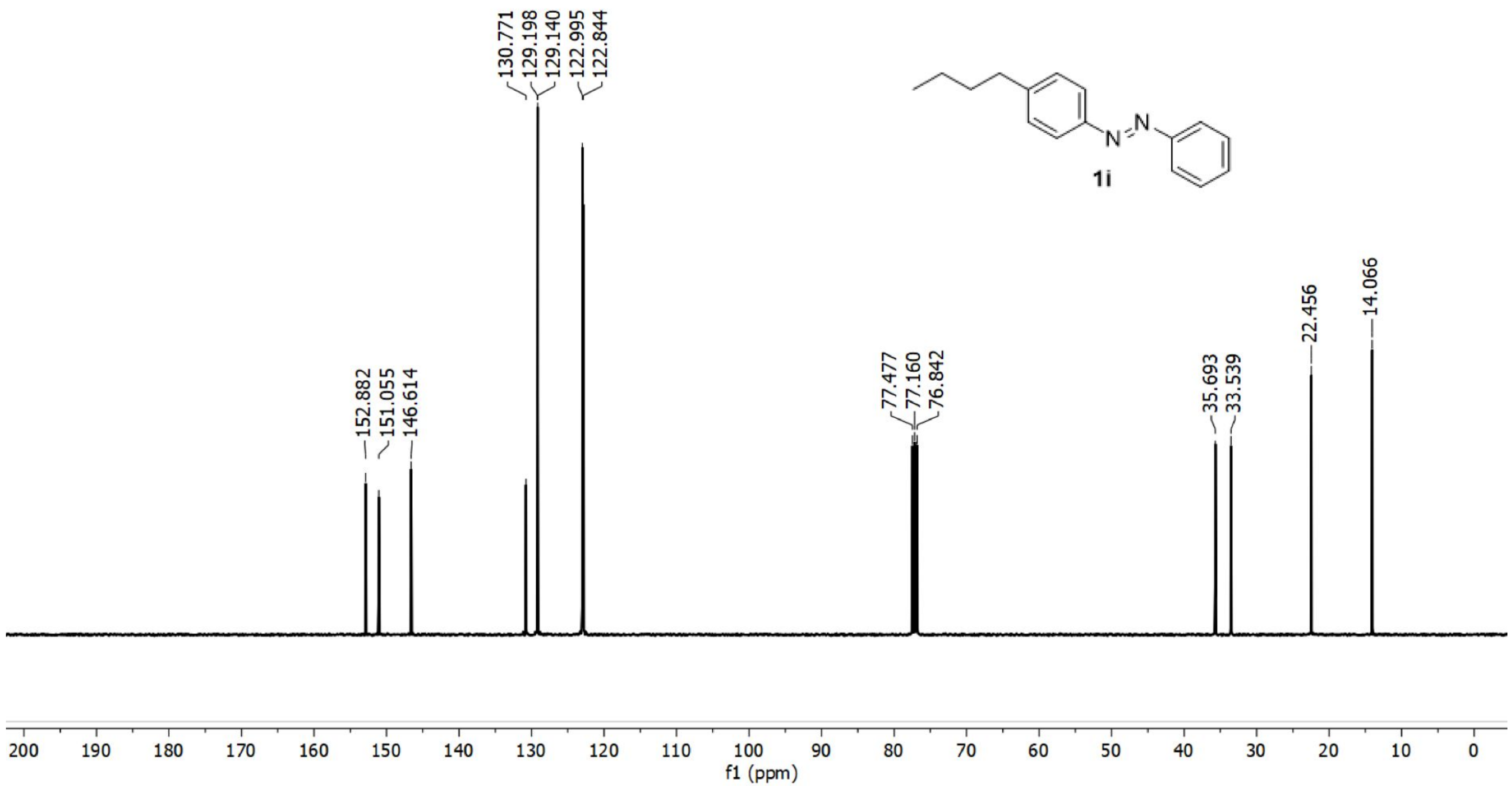

Figure S55. $^{13}C\{^{1}H\}$ NMR spectrum of (*E*)-1-(4-butylphenyl)-2-phenyldiazene (**1i**) measured in $CDCl_3$, 101 MHz, 298 K.

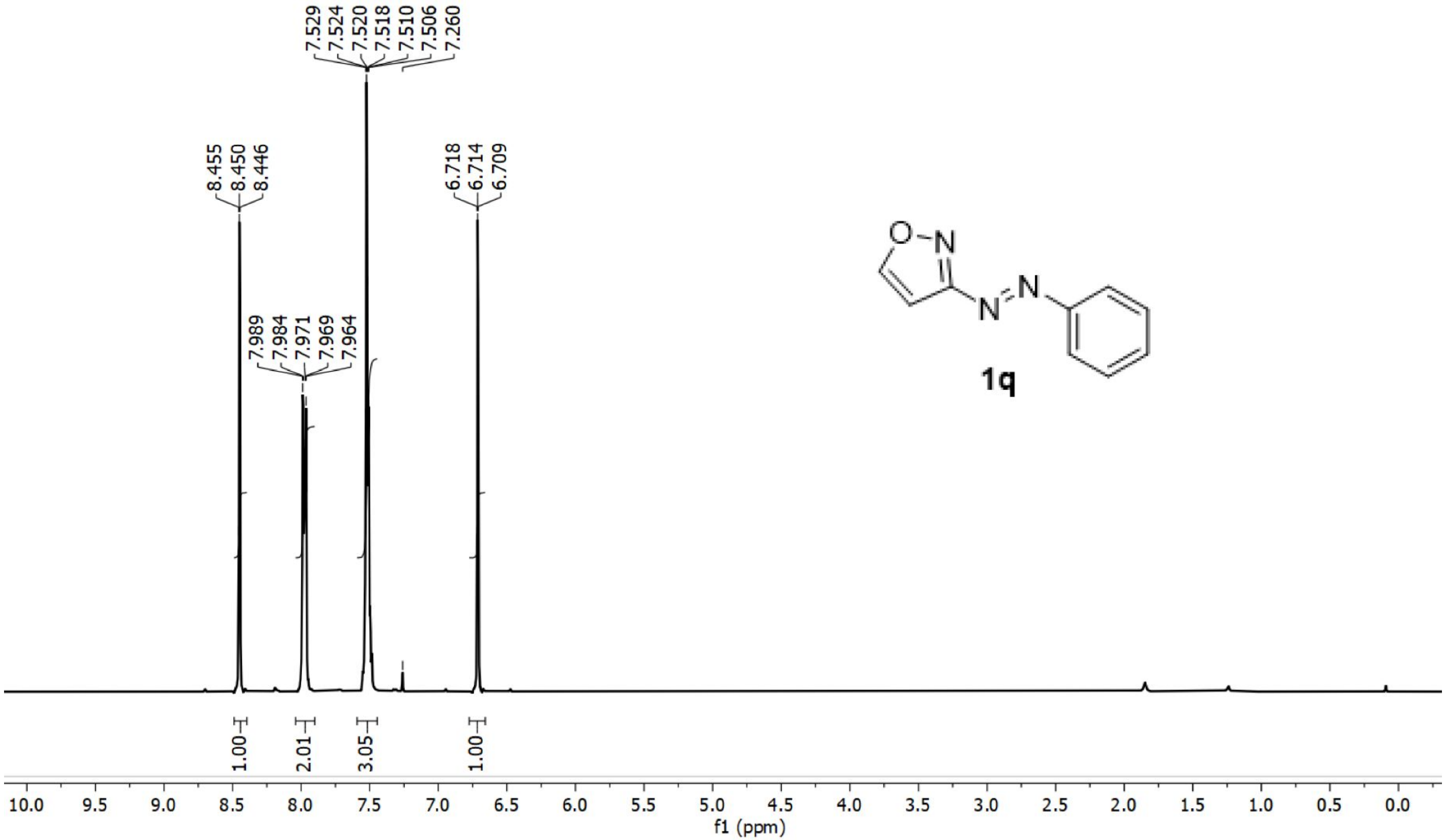

Figure S56. ^{1}H NMR spectrum of (*E*)-3-(phenyldiazenyl)isoxazole (**1q**) measured in $CDCl_3$, 400 MHz, 298 K.

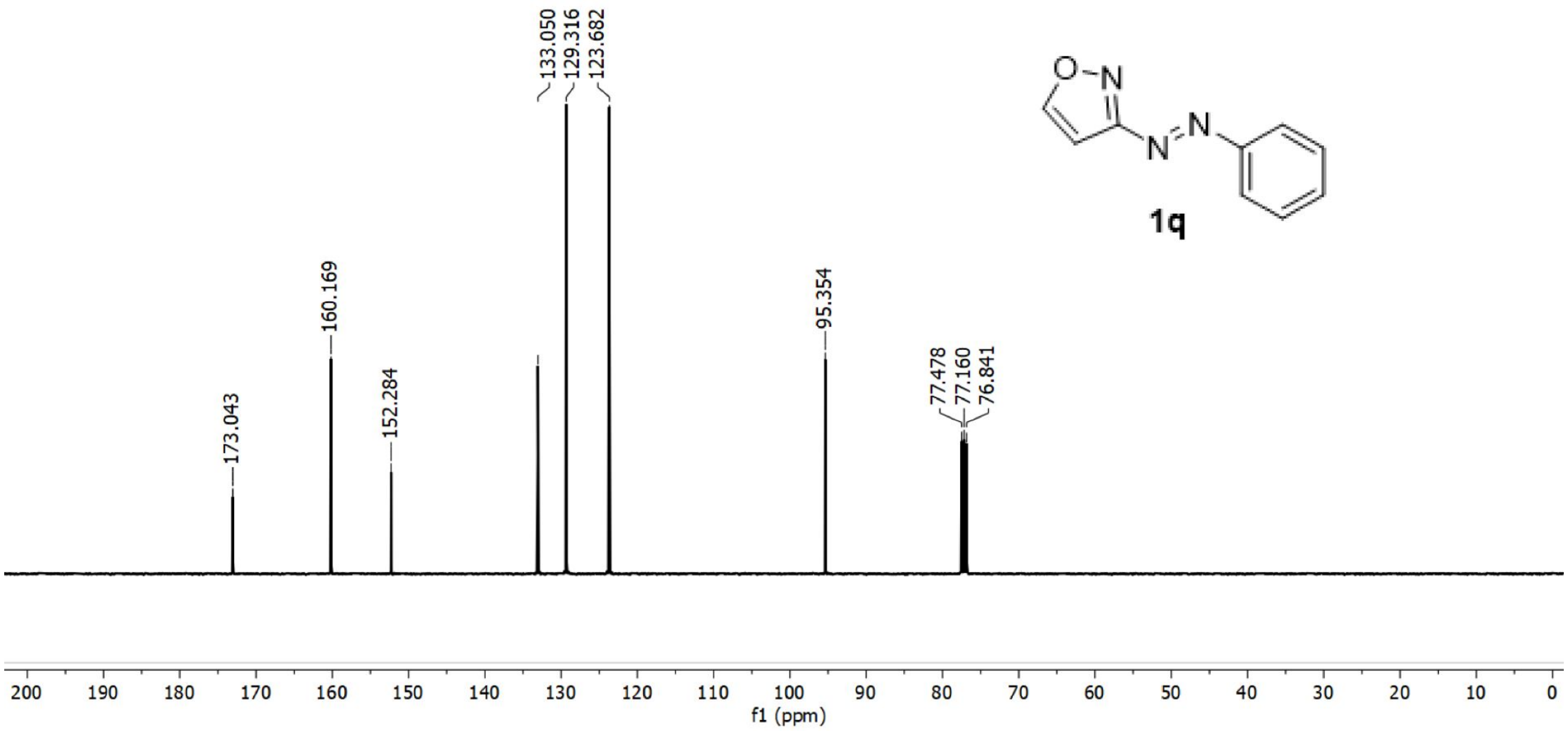

Figure S57. $^{13}C\{^{1}H\}$ NMR spectrum of (*E*)-3-(phenyldiazenyl)isoxazole (**1q**) measured in $CDCl_3$, 101 MHz, 298 K.

F. References

1. Fulmer, G. R.; Miller, A. J. ; Sherden, N. H.; Gottlieb, H. E.; Nudelman, A.; Stoltz, B. M.; Bercaw, J. E.; Goldberg, K. I. NMR Chemical Shifts of Trace Impurities: Common Laboratory Solvents, Organics, and Gases in Deuterated Solvents Relevant to the Organometallic Chemist. *Organometallics* **2010**, *29*, 2176–2179.
2. Nicholson, R. S. Theory and Application of Cyclic Voltammetry for Measurement of Electrode Reaction Kinetics. *Anal. Chem.* **1965**, *37*, 1351–1355.
3. Lavagnini, I.; Antiochia, R.; Magno, F. An Extended Method for the Practical Evaluation of the Standard Rate Constant from Cyclic Voltammetric Data. *Electroanalysis* **2004**, *16*, 505–506.
4. Lian, Y.; Bergman, R. G.; Lavis, L. D.; Ellman, J. A. Rhodium(III)-catalyzed Indazole Synthesis by C-H Bond Functionalization and Cyclative Capture. *J. Am. Chem. Soc.* **2013**, *135*, 7122–7125.
5. Zhao, W.; Zeng, X.; Huang, L.; Qiu, S.; Xie, J.; Yu, H.; Wei, Y. Oxidative Dehydrogenation of Hydrazines and Diarylamines Using a Polyoxomolybdate-Based Iron Catalyst. *Chem. Commun*. **2021**, *57*, 7677–7680.
6. Zantioti-Chatzouda, E.-M.; Malliotaki, D.; Stratakis, M. Transfer Hydrogenation of Azoarenes to Hydrazoarenes by Ammonia Borane Complex Catalyzed by Au Nanoparticles. *Adv. Synth. Catal.* **2023**, *365*, 2982–2987.
7. Lv, H.; Laishram, R. D.; Yang, Y.; Li, J.; Xu, D.; Zhan, Y.; Luo, Y.; Su, Z.; Morec, S.; Fan, B.; TEMPO Catalyzed Oxidative Dehydrogenation of Hydrazobenzenes to Azobenzenes. *Org. Biomol. Chem*. **2020**, *18*, 3471–3474.
8. Wang, Z.-Q.; Yu, J.-X.; Bai, S.-Q.; Liu, B.; Wang, C.-Y.; Li, J.-H. Oxidative Dehydrogenation of Hydrazobenzenes toward Azo Compounds Catalyzed by *tert*-Butyl Nitrite in EtOH. *ACS Omega* **2020**, *5*, 28856–28862.
9. Schönberger, M.; Althaus, M.; Fronius, M.; Clauss, W.; Trauner, D. Controlling Epithelial Sodium Channels with Light Using Photoswitchable Amilorides. *Nat. Chem*. **2014**, *6*, 712–719.
10. Powers, I. G.; Andjaba, J. M.; Luo, X.; Mei, J.; Uyeda, C. Catalytic Azoarene Synthesis from Aryl Azides Enabled by a Dinuclear Ni Complex. *J. Am. Chem. Soc.* **2018**, *140*, 4110–4118.
11. Shigeno, M.; Imamatsu, M.; Kai, Y.; Kiriyama, M.; Ishida, S.; Nozawa-Kumada, K.; Kondo, Y. Construction of 1,2,3-Benzodiazaborole by Electrophilic Borylation of Azobenzene and Nucleophilic Dialkylative Cyclization. *Org. Lett*. **2021**, *23*, 8023–8027.
12. Wang, L.; Ishida, A.; Hashidoko, Y.; Hashimoto, M. Dehydrogenation of the NH−NH Bond Triggered by Potassium tert-Butoxide in Liquid Ammonia. *Angew. Chem. Int. Ed.* **2017**, *56*, 870–873.
13. Xiong, B.; Wang, G.; Wan, L.; Xiong, T.; Zhou, C.; Liu, Y.; Zhang, P.; Yang, C.; Tang, K. Brønsted-Acid-Catalyzed *para*-Selective Diazotization of Anilines with Aryl Diazonium Tetrafluoroborates. *ChemistrySelect* **2018**, ***3***, 5147–5152.
14. Wang, X.; Wang, X.; Xia, C.; Wu, L. Visible-Light-Promoted Oxidative Dehydrogenation of Hydrazobenzenes and Transfer Hydrogenation of Azobenzenes. *Green Chem*. **2019**, *21*, 4189–4193.

15. Yi, X.; Jiao, L.; Xi, C. I_2-Mediated 2H-Indazole Synthesis *via* Halogen-Bond-Assisted Benzyl C–H Functionalization. *Org. Biomol. Chem.* **2016**, *14*, 9912–9918.
16. Bannwarth, A.; Schmidt, S. O.; Peters, G.; Sönnichsen, F. D.; Thimm, W.; Herges, R.; Tuczek, F. Fe^{III} Spin-Crossover Complexes with Photoisomerizable Ligands: Experimental and Theoretical Studies on the Ligand-Driven Light-Induced Spin Change Effect. *Eur. J. Inorg. Chem.* **2012**, *16*, 2776–2783.
17. Dougan, S. J.; Melchart, M.; Habtemariam, A.; Parsons, S.; Sadler, P. J. Phenylazo-pyridine and Phenylazo-pyrazole Chlorido Ruthenium(II) Arene Complexes: Arene Loss, Aquation, and Cancer Cell Cytotoxicity. *Inorg. Chem.* **2006**, *45*, 10882–10894.
18. Weston, C. E.; Richardson, R. D.; Haycock, P. R.; White, A. J. P.; Fuchter, M. J. Arylazopyrazoles: Azoheteroarene Photoswitches Offering Quantitative Isomerization and Long Thermal Half-Lives. *J. Am. Chem. Soc.* **2014**, *136*, 11878–11881.
19. Faustino, H.; Brannigan, C. R.; Reis, L.V.; Santos, P. F.; Almeida, P. Novel Azobenzothiazole Dyes from 2-Nitrosobenzothiazoles. *Dyes and Pigments* **2009**, *83*, 88–94.
20. Glotz, G.; Knaipp, K.; Maier, M. S.; Hüll, K.; Novak, A.; Kelterer, A.-M.; Griebenow, T.; Herges, R.; Trauner, D.; Gescheidt, G. To Isomerize or not to Isomerize? E/Z Isomers of Cyclic Azobenzene Derivatives and Their Reactivity Upon One Electron Reduction. *Chem. Eur. J.* **2023**, *29*, e202300146.
21. Wang, X.; Chai, J.; Lashgari, A.; Jiang, J. J. Azobenzene-Based Low-Potential Anolyte for Nonaqueous Organic Redox Flow Batteries. *ChemElectroChem* **2021**, *8*, 83–89.
